# Interface-Tailored Secondary Excitation and Ultrafast Charge/Energy Transfer in Ti_3_C_2_T_*x*_-MoS_2_ Heterostructure Films

**DOI:** 10.1021/jacs.5c01826

**Published:** 2025-03-07

**Authors:** Jiaxu Zhang, Rafael Muñoz-Mármol, Shuai Fu, Xiaodong Li, Wenhao Zheng, Andrea Villa, Giuseppe M. Paternò, Darius Pohl, Alexander Tahn, Mike Hambsch, Stefan C. B. Mannsfeld, Dongqi Li, Hao Xu, Quanquan Guo, Hai I. Wang, Francesco Scotognella, Minghao Yu, Xinliang Feng

**Affiliations:** † Faculty of Chemistry and Food Chemistry & Center for Advancing Electronics Dresden (cfaed), Technische Universität Dresden, 01062 Dresden, Germany; ‡ Instituto Universitario de Materiales, University of Alicante, 03690 San Vicente del Raspeig, Spain; § Department of Physics, Politecnico di Milano, 20133 Milan, Italy; ∥ Max Planck Institute of Microstructure Physics, 06120 Halle (Saale), Germany; ⊥ Max Planck Institute for Polymer Research, 55128 Mainz, Germany; # Center for Nanoscience and Technology, Istituto Italiano di Tecnologia, 20134 Milano, Italy; ∇ Dresden Center for Nanoanalysis (DCN), Dresden, Center for Advancing Electronics Dresden (cfaed), TUD Dresden University of Technology, 01062 Dresden, Germany; ○ Center for Advancing Electronics Dresden (cfaed) & Faculty of Electrical and Computer Engineering, TUD Dresden University of Technology, 01062 Dresden, Germany; ◆ Nanophotonics, Debye Institute for Nanomaterials Science, Utrecht University, Princetonplein 1, 3584 CC Utrecht, The Netherlands; ¶ Department of Applied Science and Technology, Politecnico di Torino, Corso Duca degli Abruzzi 24, 10129 Torino, Italy

## Abstract

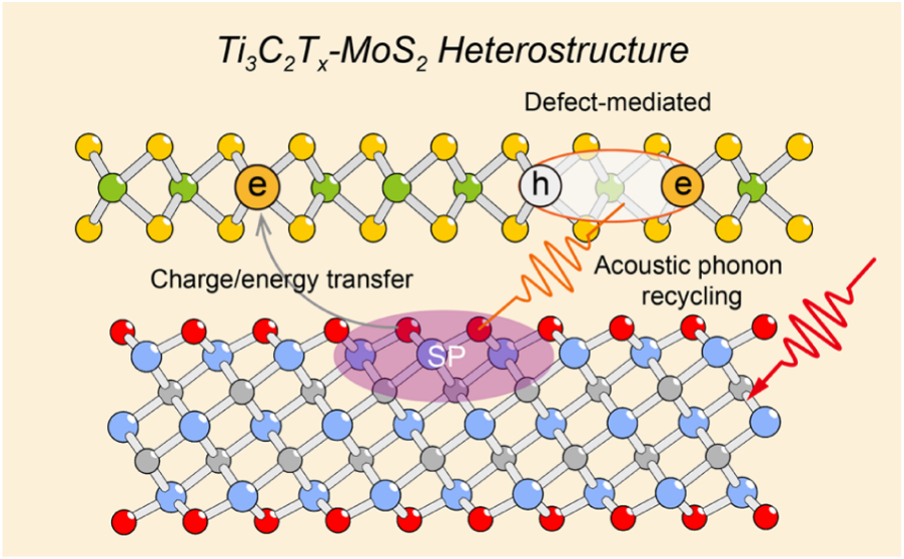

Charge/energy separation across interfaces of plasmonic materials is vital for minimizing plasmonic losses and enhancing their performance in photochemical and optoelectronic applications. While heterostructures combining plasmonic two-dimensional transition metal carbides/nitrides (MXenes) and semiconducting transition metal dichalcogenides (TMDs) hold significant potential, the mechanisms governing plasmon-induced carrier dynamics at these interfaces remain elusive. Here, we uncover a distinctive secondary excitation phenomenon and an ultrafast charge/energy transfer process in heterostructure films composed of macro-scale Ti_3_C_2_T_*x*_ and MoS_2_ films. Using Rayleigh–Bénard convection and Marangoni effect-induced self-assembly, we fabricate large-scale (square centimeters) Ti_3_C_2_T_*x*_ and MoS_2_ films composed of edge-connected monolayer nanoflakes. These films are flexibly stacked in a controlled sequence to form macroscopic heterostructures, enabling the investigation and manipulation of excited-state dynamics using transient absorption and optical pump-terahertz probe spectroscopy. In the Ti_3_C_2_T_*x*_-MoS_2_ heterostructure, we observe a secondary excitation in MoS_2_ driven by the surface plasmon resonance of Ti_3_C_2_T_*x*_. This phenomenon, with a characteristic rise time constant of ∼70 ps, is likely facilitated by acoustic phonon recycling across the interface. Further interfacial thermal transport engineering—achieved by tailoring the sequence and combination of interfaces in trilayer heterostructures—allows extending the characteristic time to ∼175 ps. Furthermore, we identify a sub-150 fs ultrafast charge/energy transfer process from Ti_3_C_2_T_*x*_ to MoS_2_. The transfer efficiency is strongly dependent on the excitation photon energy, resulting in amplified photoconductivity in MoS_2_ by up to ∼180% under 3.10 eV excitation. These insights are crucial for developing plasmonic MXene-based heterostructures, paving the way for advancements in photochemical and optoelectronic applications.

## Introduction

Plasmonic materials are well-known for their ability to confine photoenergy through surface plasmon resonances or localized surface plasmon resonance, making them highly promising for applications in photochemical reactions and optoelectronic devices.^1−3^ Recently, emerging two-dimensional (2D) transition metal carbides/nitrides (i.e., MXenes with Ti_3_C_2_T_*x*_ as the representative case) have been revealed with a wide array of surface plasmon (SP) modes across the visible, near-infrared, and mid-infrared wavelengths.^4−6^ Typically, MXenes are synthesized by selectively etching atomic layers from their MAX precursors, sharing the general formula of M_*n*+1_X*_n_*T_*x*_ (M represents a transition metal, X denotes C and/or N, and T_*x*_ refers to surface terminations).^7^ They exhibit remarkable structural diversity, including variations in M and X elements, atomic layer numbers, in-plane and out-of-plane ordering, solid solution lattices, and abundant surface chemistries.^7−9^ This structural diversity offers a vast playground for SP engineering,^10,11^ positioning MXenes as a unique class of plasmonic materials with diverse applications. For instance, plasmon-induced thermalization and hot-electron injection in the visible and near-infrared region have been shown to significantly enhance the electrocatalytic activity of MXenes in the hydrogen evolution reaction across a broad pH range, boosting the catalytic performance by more than 5-fold.^12^ Moreover, Mo_2_CT_*x*_ MXene-based plasmonic photodetectors demonstrated high responsivity (up to 9 A W^–1^) and detectivity (≈ 5 × 10^11^ Jones), outperforming many other 2D materials due to intrinsic plasmon-assisted hot carrier generation.^13^

However, plasmon-generated hot carriers face rapid kinetic energy dissipation as lattice heat, which significantly limits their effectiveness in potential applications.^14^ In MXenes, this dissipation pathway has been proposed to involve multiple energy conversion processes, including direct plasmon-phonon coupling, nonthermal electron–phonon coupling after Landau damping, and thermal electron–phonon coupling following electron thermalization.^15^ Time-resolved spectroscopy and ultrafast electron diffraction have provided important insights into ultrafast hot carrier-lattice relaxation in MXenes, observing carrier-phonon scattering within 100 fs^15,16^ and excited lattice vibrations within approximately 230 fs,^17^ respectively. Constructing an interface that enables ultrafast charge separation and energy transfer represents a promising pathway to manipulate plasmon-vibration interactions and mitigate plasmonic losses. In this context, heterostructures comprising MXenes and semiconducting transition metal dichalcogenides (TMDs) are of great interest owing to the intimate contact at the 2D–2D interfaces and the strong light-matter interactions in TMDs.^18^ As a classic example, hybrid Ti_3_C_2_T_*x*_/MoS_2_ plasmonic photodetectors have demonstrated greatly enhanced performance, achieving a 150-fold increase in detectivity compared to pristine MoS_2_, reaching 2.33 × 10^12^ Jones under 635 nm illumination.^19^ Nevertheless, the fundamental interfacial charge/energy dynamics in MXene-TMD heterostructures, essential for guiding future interface optimization, has remained elusive. A major challenge arises from the limited size of flake-stacked MXene-TMD interfaces, as the top-down etching-delamination synthesis limits MXene nanoflakes to the micrometer range,^20^ restricting direct probing of interfacial dynamics via conventional spectroscopic methods. Alternatively, heterostructures formed through solution processing or coating techniques may compromise interface control, introducing extrinsic properties and interferences that impede an understanding of the intrinsic properties.

**Figure 1:**
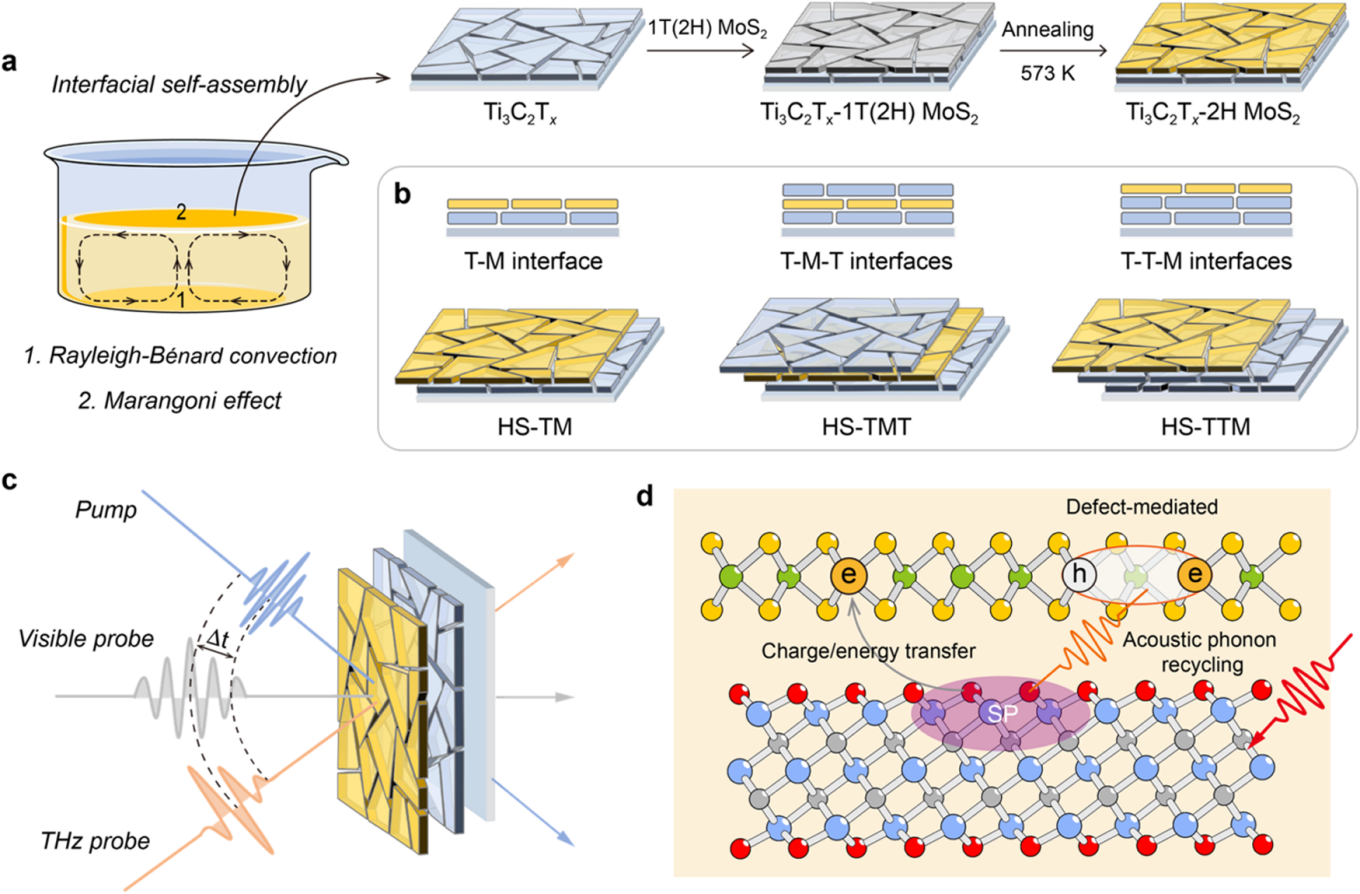
Schematics illustrating the preparation of Ti_3_C_2_T_*x*_-MoS_2_ heterostructure films and their excited-state dynamics. (a) Interfacial self-assembly method for fabricating macro-scale Ti_3_C_2_T_*x*_ and MoS_2_ films. The heterostructure films are constructed by stacking the MoS_2_ film on top of the Ti_3_C_2_T_*x*_ film, followed by an annealing process to completely convert MoS_2_ into the semiconducting 2H phase. (b) Schematic representation of three distinct Ti_3_C_2_T_*x*_-MoS_2_ heterostructure configurations: HS-TM, HS-TMT, and HS-TTM. (c) Schematic of time-resolved pump–probe measurements employed to investigate the excited-state dynamics. (d) Schematic showing an ultrafast charge/energy transfer from Ti_3_C_2_T_*x*_ to MoS_2_, followed by a secondary excitation in MoS_2_ driven by the SP excitation of Ti_3_C_2_T_*x*_.

In this study, we employ Ti_3_C_2_T_*x*_ and MoS_2_ as model materials to construct macro-scale heterostructure films, uncovering a unique secondary excitation mechanism and an ultrafast charge/energy transfer process within the heterostructure. Specifically, we fabricate individual Ti_3_C_2_T_*x*_ and MoS_2_ films composed of edge-connected monolayer nanoflakes with macroscopically continuous areas (up to square centimeters) by leveraging Rayleigh–Bénard convection and Marangoni effect-induced self-assembly (Figure 1a).^21,22^ The flexible stacking of these films allows the construction of macro-scale heterostructure films with tunable configurations (Figure 1b), enabling direct detection and control of excited-state dynamics through transient absorption spectroscopy and optical pump-terahertz (THz) probe spectroscopy (Figure 1c). In a bilayer Ti_3_C_2_T_*x*_-MoS_2_ heterostructure film (denoted HS-TM), we discover an intriguing secondary excitation of MoS_2_ under 1.55 eV excitation, where the SP resonance of Ti_3_C_2_T_*x*_ dominates the interfacial dynamics. This phenomenon is explained by acoustic phonon recycling with a characteristic time of ∼70 ps, where heat generated in Ti_3_C_2_T_*x*_ transfers to MoS_2_ and activates electronic excitation (Figure 1d). We also construct trilayer heterostructure films with configurations of Ti_3_C_2_T_*x*_*-*MoS_2_-Ti_3_C_2_T_*x*_ (denoted HS-TMT) and Ti_3_C_2_T_*x*_-Ti_3_C_2_T_*x*_-MoS_2_ (HS-TTM), demonstrating how interface engineering influences interfacial thermal transport and extends the excited-state lifetime in MoS_2_. Notably, HS-TTM shows a long-lived excited-state lifetime exceeding 800 ps, significantly longer than the MoS_2_ film (92 ps), HS-TM (341 ps), and HS-TMT (550 ps). Additionally, we unveil that the ultrafast charge/energy transfer from Ti_3_C_2_T_*x*_ to MoS_2_ occurs within 150 fs. This transfer exhibits a strong dependence on the excitation photon energy, with interband transition (IBT) excitation of Ti_3_C_2_T_*x*_ amplifying the photoconductivity of HS-TM by up to ∼180% compared to individual MoS_2_ under an equivalent absorbed photon density.

## Results and Discussion

### Preparation of Ti_3_C_2_T_*x*_-MoS_2_ Heterostructure Films

**Figure 2:**
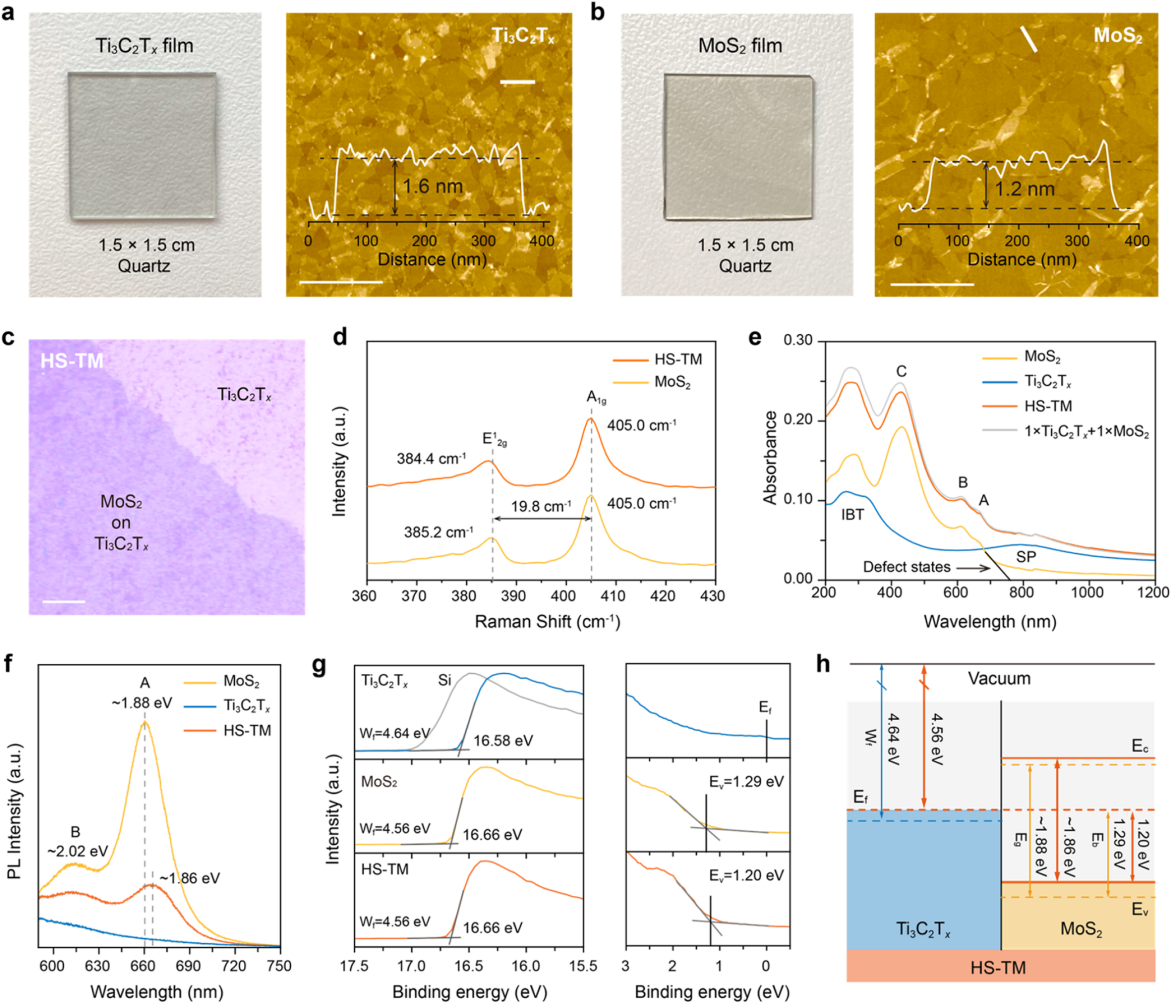
Characterizations of Ti_3_C_2_T_*x*_-MoS_2_ heterostructures. Optical and AFM images along with thickness profiles of macro-scale (a) Ti_3_C_2_T_*x*_ film and (b) MoS_2_ film (scale bars, 1 μm). (c) Optical microscopy image of HS-TM (scale bar, 20 μm). (d) Raman spectra of the MoS_2_ film and HS-TM. (e) Steady-state UV–vis–NIR absorption, (f) PL, and (g) UPS spectra of the Ti_3_C_2_T_*x*_ film, the MoS_2_ film, and HS-TM. (h) Schematic showing band alignment in HS-TM.

We first adopted a self-assembly approach to fabricate individual macro-scale Ti_3_C_2_T_*x*_ and MoS_2_ films using their respective nanoflakes. Aqueous dispersions of Ti_3_C_2_T_*x*_ nanoflakes (Figure S1) and MoS_2_ nanoflakes (Figure S2) were prepared and diluted in glass beakers. Ethyl acetate was then added dropwise to the surface of each dispersion, inducing Rayleigh–Bénard convection during evaporation. This convection, combined with the high surface tension of water, compactly assembled the nanoflakes on the water surface due to the Marangoni effect, forming continuous films that covered the whole liquid surface.^21,22^ Using this method, we achieved continuous Ti_3_C_2_T_*x*_ and MoS_2_ films with areas extending up to square centimeters. These films can be easily transferred onto any desired substrates including but not limited to silicon or quartz plates via a “fishing” step for further processing. Optical microscopy images verify the macroscopic homogeneity of both films (Figure S3). Atomic force microscopy (AFM) images reveal that both Ti_3_C_2_T_*x*_ (Figure 2a) and MoS_2_ (Figure 2b) films consist predominantly of monolayer flakes with slight overlap at the edges of adjacent flakes, achieving nanoflake coverage of 89.3 and 95.3%, respectively (Figure S4). These individual films can be easily stacked to create macro-scale, face-to-face Ti_3_C_2_T_*x*_-MoS_2_ heterostructure films, where the underlying Ti_3_C_2_T_*x*_ film maintains good coverage and morphological stability (Figure S5). To demonstrate the robustness and ease of this process, we created a multilayer heterostructure film by alternately stacking four Ti_3_C_2_T_*x*_ layers and three MoS_2_ layers, resulting in a clearly visible layered architecture (Figure S6).

Our emphasis then shifted to HS-TM, which was obtained by stacking a single MoS_2_ layer onto a Ti_3_C_2_T_*x*_ layer (Figures 2c and S7). All films were annealed at 300 °C under an Ar atmosphere to eliminate residual solvent moisture and ensure the complete transition of MoS_2_ to the semiconducting 2H phase (Figures S8 and S9).^23^ Both MoS_2_ and Ti_3_C_2_T_*x*_ films show good chemical and morphological stability after thermal treatment (Figures S9 and S10). Raman spectra unveiled a redshift in the E_2g_^1^ phonon mode of MoS_2_ (from 385.2 cm^–1^ in the MoS_2_ film to 384.4 cm^–1^ in HS-TM), which can be attributed to stacking-induced changes in intralayer bonding and/or Coulombic interlayer interactions (Figure 2d).^24^ Besides, grazing-incidence wide-angle X-ray scattering (GIWAXS) revealed interfacial interactions in HS-TM, evidenced by a shift of the (100) diffraction compared to MoS_2_. This suggests an in-plane expansion of the lattice of the heterostructure compared to the individual materials possibly due to lattice matching (Figure S11).

We collected steady-state ultraviolet–visible–near-infrared (UV–vis–NIR) absorption spectra for the Ti_3_C_2_T_*x*_ film, MoS_2_ film, and HS-TM (Figure 2e). The Ti_3_C_2_T_*x*_ film exhibited a broad peak centered around 800 nm, characteristic of its transversal SP mode,^5,11^ along with two ultraviolet peaks at approximately 260 and 325 nm, corresponding to its IBTs.^11,25^ Meanwhile, the MoS_2_ film showed the characteristic A exciton (667 nm), B exciton (611 nm), and C exciton (430 nm) peaks associated with 2H-phase MoS_2_. A subresonant absorption tail was also observed, attributed to the presence of atomic defects.^26,27^ Moreover, the spectrum of HS-TM retained all the characteristic absorption peaks of the individual Ti_3_C_2_T_*x*_ and MoS_2_ films. The superposition of the spectra of Ti_3_C_2_T_*x*_ and MoS_2_ in equal proportions (i.e., 1 × Ti_3_C_2_T_*x*_ + 1 × MoS_2_) closely matches the spectrum of HS-TM. Figure 2f compares the photoluminescence (PL) spectra of the three films under 488 nm excitation. The MoS_2_ film displayed evident peaks at 660 nm (1.88 eV) and 613 nm (2.02 eV), corresponding to the radiative emissions of A and B excitons of 2H-phase MoS_2_, respectively. In HS-TM, a redshift in exciton resonances, along with reduced PL intensity, was observed. This behavior can be attributed to two factors: (1) changes in Coulomb interactions potentially induced by alterations in the dielectric environment or strong screening effects by the free carriers in MXenes,^28^ or (2) the presence of ultrafast interfacial processes.^29^ The band structure evolution of MoS_2_ in the Ti_3_C_2_T_*x*_-MoS_2_ heterostructure was further corroborated by density functional theory calculations, suggesting a potential transition from a direct to an indirect bandgap (Figure S12).

Furthermore, ultraviolet photoelectron spectroscopy (UPS) was conducted to assess the band alignment of Ti_3_C_2_T_*x*_ and MoS_2_ (Figure 2g). The Ti_3_C_2_T_*x*_ film showed a secondary electron cutoff energy (*E*_cutoff_) of ∼16.58 eV, from which its work function (*W*_F_) was estimated as ∼4.64 eV by subtracting the He I excitation energy (21.22 eV). For the MoS_2_ film, the *E*_cutoff_ was measured at ∼16.66 eV, corresponding to a *W*_F_ of ∼4.56 eV. The valence band maximum (*E*_v_) of MoS_2_ was found to be around 1.29 eV below the Fermi level (*E*_F_). Given its bandgap of ∼1.88 eV, *E*_F_ resides close to the conduction band minimum (*E*_c_), indicating the n-type nature of the MoS_2_ film, presumably originating from sulfur vacancies.^30,31^ The *W*_F_ of HS-TM was measured to be approximately 4.56 eV, similar to the MoS_2_ film. However, the energy difference between *E*_v_ and *E*_F_ narrowed to ∼1.20 eV in HS-TM. These results indicate that both *E*_v_ and *E*_c_ of MoS_2_ in the heterostructure shift toward the vacuum energy level compared to the individual MoS_2_ film (as illustrated in Figure 2h), revealing strong interactions between Ti_3_C_2_T_*x*_ and MoS_2_. Although the *E*_F_ of MoS_2_ in the heterostructure shows minimal change compared to the MoS_2_ film, its position relative to *E*_v_ is closer, suggesting electron transfer from MoS_2_ to Ti_3_C_2_T_*x*_. This electronic doping effect, narrowing the gap between *E*_F_ and *E*_v_, was further corroborated by Mo 3d and S 2p X-ray photoelectron spectroscopy (XPS) spectra, which showed lowered binding energies in HS-TM (Figure S9).^32,33^

### Secondary Excitation and Prolonged Excited States

**Figure 3:**
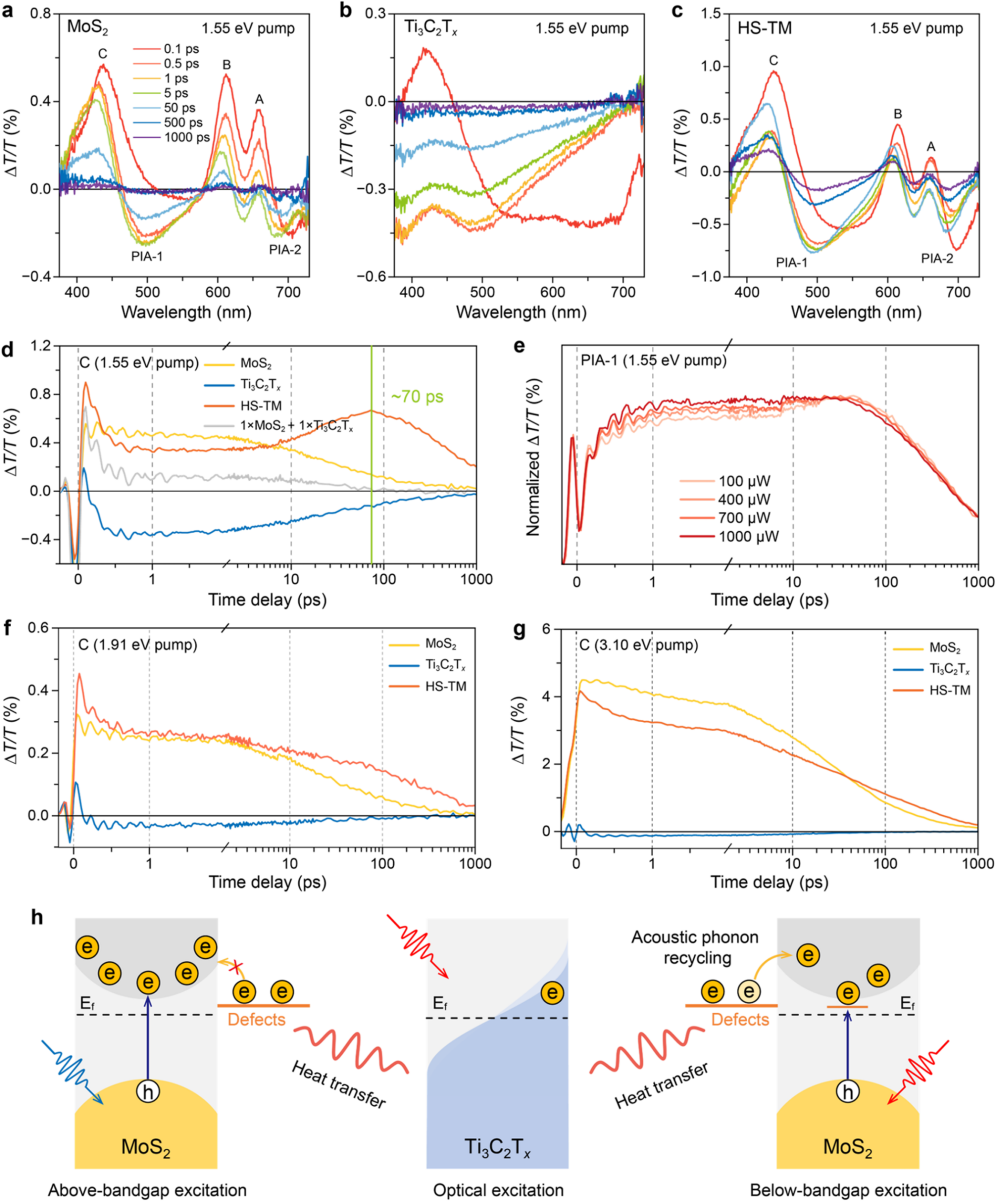
TA spectra and excited-state dynamics. TDT spectra of (a) the MoS_2_ film, (b) the Ti_3_C_2_T_*x*_ film, and (c) HS-TM at 0.1–1000 ps under 1.55 eV excitation with 70 fs-width pulses and 1.2 mJ cm^–2^ fluence. (d) TA kinetics pumped at 1.55 eV and probed at 436 nm (C exciton bleaching) of the MoS_2_ film, the Ti_3_C_2_T_*x*_ film, HS-TM, and the superposed curve of the MoS_2_ film and the Ti_3_C_2_T_*x*_ film (i.e., 1 × MoS_2_ + 1 × Ti_3_C_2_T_*x*_). (e) Pump fluence-dependent PIA-1 (494 nm) kinetics of HS-TM pumped at 1.55 eV. TA kinetics curves of the MoS_2_ film, the Ti_3_C_2_T_*x*_ film, and HS-TM at C exciton bleaching pumped at (f) 1.91 eV and (g) 3.10 eV. (h) Schematic illustrating the mechanism governing the secondary excitation in the Ti_3_C_2_T_*x*_-MoS_2_ heterostructure.

The macro-scale heterostructure films enable direct probing of their electronic excitations using transient absorption (TA) spectroscopy. We first employed a 1.55 eV (800 nm) optical pump pulse to activate the SP excitation of Ti_3_C_2_T_*x*_ and investigated the excited-state dynamics of the Ti_3_C_2_T_*x*_ film, the MoS_2_ film, and HS-TM. Transient differential transmittance (TDT) spectra were retrieved by measuring the differential transmission signals with and without photoexcitation (Δ*T*/*T =* [*T*_withpump_ – *T*_w/opump_]/*T*_w/opump_). As exhibited in Figure 3a, the MoS_2_ film exhibits a characteristic optical response following optical excitations mediated by defects. In this process, optical excitations via defect states (see static absorption in Figure 2e) promote trapped electrons (holes) from defect states to the conduction (valence) band of MoS_2_.^26,27^ This electronic occupation enhances the screening effect, causing simultaneous renormalization of the electronic gap and excitonic binding energy, which tend to compensate for each other.^34^ This many-body effect results in the simultaneous bleaching of the A exciton (660 nm), B exciton (616 nm) and C exciton (436 nm) peaks of MoS_2_. Additionally, this effect is reflected by a red-shifted photoinduced absorption (PIA-1 (494 nm) and PIA-2 (684 nm)) within 0.5 ps, followed by blue-shifted excitonic transitions. In contrast, the TDT spectrum of the Ti_3_C_2_T_*x*_ film displays a bleaching peak at ∼424 nm and a PIA band covering the entire spectrum at 100 fs, which later evolves into a broad PIA band with a maximum near 500 nm (Figure 3b). This behavior aligns with a thermalization scenario in which hot carriers with an undefined electronic distribution rapidly evolve into thermalized carriers following the Fermi–Dirac distribution.^35^

The TDT spectra of HS-TM are characterized by apparent excitonic peaks associated with MoS_2_, along with more pronounced PIA bands resulting from the combined response of both MoS_2_ and Ti_3_C_2_T_*x*_ (Figure 3c). Intriguingly, the C exciton bleaching shows an initial intensity decay within the first 1 ps, followed by a remarkable “turnaround” feature characterized by sustained growth lasting up to 70 ps (Figure 3d). This “turnaround” feature symbolizes secondary excitation of MoS_2_ in HS-TM, leading to a prolonged excited-state lifetime, contrasting with the monotonic decay observed in the MoS_2_ film. Of note, the kinetics and spectral features of HS-TM are distinctly different from the individual signals of the MoS_2_ film and the Ti_3_C_2_T_*x*_ film, as well as their superposition (i.e., 1 × MoS_2_ + 1 × Ti_3_C_2_T_*x*_) (Figures 3d and S13), indicating that the negative background generated by the PIA of Ti_3_C_2_T_*x*_ is not the reason for the observed “turnaround” feature in HS-TM. Likewise, the kinetics curve of PIA-1 in HS-TM also displayed a “turnaround” feature, with an onset at 8 ps (Figure S14). This feature is in stark contrast with the monotonic decay of the negative signals observed in both the MoS_2_ film and the Ti_3_C_2_T_*x*_ film after 10 ps. To further explore this “turnaround” feature, pump fluence-dependent experiments were performed. The normalized dynamics curves of PIA-1 revealed that the signal decay within the first 2 ps was associated with the pump fluence (Figure 3e). The oscillation feature, agreeing well with the previous observation in Ti_3_C_2_T_*x*_ (Figure S15), was attributed to direct energy transfer from nonthermal electrons to out-of-plane A_1g_ (∼60 fs) and in-plane E_g_ coherent phonons (1–2 ps) following SP excitation and Landau damping.^15^ This observation suggests that the onset of the “turnaround” feature follows or overlaps electron–phonon coupling.

Previous spectroscopic studies have shown that ultrafast photoexcitation can transiently heat the electron bath of Ti_3_C_2_T_*x*_, followed by ultrafast subps carrier-lattice relaxation driven by efficient electron–phonon interactions in Ti_3_C_2_T_*x*_.^15,16^ Moreover, considering the time scale of electron/energy transfer of Ti_3_C_2_T_*x*_,^36^ direct nonthermal electron transfer occurs within 50 fs, while nonthermal energy transfer via interface scattering takes place within 125 fs. Additionally, thermal electron transfer following electron–electron scattering emerges around 75 fs and peaks at approximately 125 fs. Consequently, the hot carrier injection scenario cannot adequately account for the observed “turnaround” feature in HS-TM happening in tens of ps following light excitations. Here we attribute this “turnaround” feature to an acoustic phonon recycling process (see follow-up details). In this process, after optical phonon emission and optical-acoustic phonon coupling, the acoustic phonons populated in Ti_3_C_2_T_*x*_ can transfer energy to MoS_2_ via thermal excitation,^37−39^ likely by promoting electrons from shallow defect states into the conduction band. A similar phenomenon was observed in graphene-WS_2_ heterostructures, where acoustic phonon recycling occurred with a characteristic time exceeding 100 ps.^39^

To further investigate the proposed acoustic phonon recycling process, pump-photon-energy dependent TA measurements were performed using optical pumps at 1.91 eV (650 nm) and 3.10 eV (400 nm). The 1.91 eV excitation matches well with the excitonic resonance in MoS_2_, while the SP excitation of Ti_3_C_2_T_*x*_ is relatively weak compared to the 1.55 eV excitation (Figure S16). Under 1.91 eV excitation, the kinetics curve of HS-TM depicted only a slightly prolonged excited-state lifetime compared to the MoS_2_ film, without displaying the pronounced “turnaround” feature (Figure 3f). In contrast, the 3.10 eV excitation induced both the IBT excitation of Ti_3_C_2_T_*x*_ and the above-bandgap excitation of MoS_2_ (Figure S17). At the C exciton bleaching, the kinetics were primarily influenced by the behavior of MoS_2_, with the “turnaround” feature and extended excited-state lifetime being less prominent (Figure 3g). Considering that Ti_3_C_2_T_*x*_ exhibits similar acoustic phonon conditions following nonthermal electron-vibration coupling and optical phonon decay, regardless of the pump excitation wavelength,^15,40,41^ the observed variation in the significance of the “turnaround” phenomenon likely stems from differences in the excited-state populations of MoS_2_ under different photon energies. Specifically, below-bandgap, defect-assisted excitation generates a significantly lower excited-state population than resonant or above-bandgap excitation. This results in a larger proportion of unoccupied electronic states, which facilitates electronic transitions driven by acoustic phonon recycling, thereby enhancing the prominence of the secondary excitation feature (Figure 3h).

### Interface-Manipulated Dynamics

**Figure 4:**
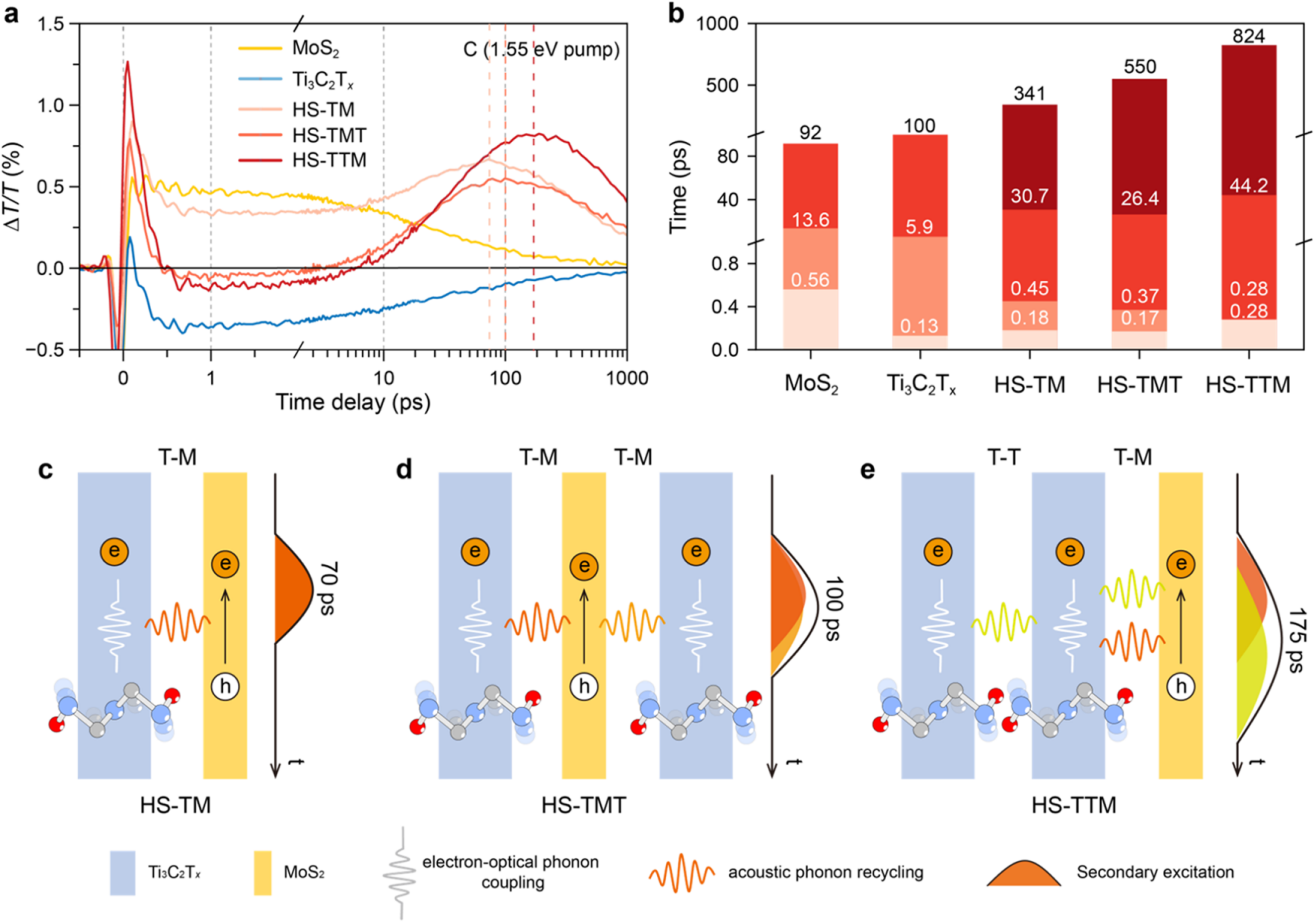
Interface-manipulated dynamics. (a) TA kinetics probed at 436 nm (C exciton bleaching) under excitation at 1.55 eV, and (b) the corresponding exponentially fitted time constants. Schematics illustrating the mechanisms governing the interfacial thermal conductivity in (c) HS-TM, (d) HS-TMT, and (e) HS-TTM. The contribution of each Ti_3_C_2_T_*x*_ layer to the secondary excitation of MoS_2_ was schematically deconvoluted and illustrated using different colors.

With the tunable stacking configurations of the macro-scale Ti_3_C_2_T_*x*_ and MoS_2_ films, we further constructed trilayer heterostructure films with varying stacking orders, namely HS-TMT and HS-TTM. In the TDT spectra obtained using 1.55 eV optical pump (Figure S18), we observed that adding an additional layer of Ti_3_C_2_T_*x*_ did not qualitatively alter the dominant optical response of MoS_2_ in HS-TMT and HS-TTM. However, we detected an enhanced negative background due to the Ti_3_C_2_T_*x*_ layers, which was particularly pronounced in HS-TTM. This effect can be attributed to the increased SP effect from the additional layer of Ti_3_C_2_T_*x*_.^11^ Moreover, the apparent “turnaround” feature, marked by a prolonged and sustained increase, was observed for the C exciton bleaching of MoS_2_ in both HS-TMT (∼100 ps) and HS-TTM (∼175 ps) (Figure 4a). To gain deeper understanding of the transformation of excited states in these films, we performed multiexponential fitting of the C exciton kinetics, using a phenomenological model based on eq 1, where *A*_*i*_ and τ_*i*_ denote the fractional amplitude and decay constant, respectively. Figure 4b illustrates the kinetics decay components for all films under 1.55 eV excitation. Three decay components were identified for the MoS_2_ film, including a fast component (τ_1_ ∼ 560 fs) typically associated with charge trapping, an intermediate component (τ_2_ ∼ 13.6 ps) related to exciton–phonon scattering and exciton–exciton annihilation, and a slow component (τ_3_ ∼ 92 ps) corresponding to exciton recombination.^42^ Meanwhile, kinetics fitting of the Ti_3_C_2_T_*x*_ film revealed three decay components, including a rapid component (τ_1_ ∼ 130 fs) attributed to electron thermalization (electron–electron scattering), a subsequent electron cooling (electron–phonon scattering) process (τ_2_ ∼ 6 ps), and a phonon diffusion process determined by the thermal boundary resistance between Ti_3_C_2_T_*x*_ and the substrate (τ_3_ ∼ 100 ps).^6,41^ The decay behavior of all three heterostructure films (HS-TM, HS-TMT, and HS-TTM) was more complex than that of the individual MoS_2_ film and the Ti_3_C_2_T_*x*_ film, with each exhibiting four decay components. Notably, the long-lived process, attributed to exciton recombination in MoS_2_,^42^ displayed significantly extended time constants in the heterostructure films. In HS-TM, the time constant increased to 341 ps, compared to 92 ps in the MoS_2_ film. This duration was further extended to 550 ps in HS-TMT and reached 824 ps in HS-TTM.
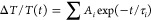
1

In addition, we conducted the TA measurements on HS-TMT and HS-TTM using 1.91 and 3.10 eV pump lights (Figure S19). Under 1.91 eV excitation, the “turnaround” feature in the C exciton kinetics became weakened but remained discernible across all heterostructure films (Figure S20). The long-lived process exhibited an especially extended time constant in HS-TTM, reaching 858 ps. Under 3.10 eV excitation, the “turnaround” feature was nearly absent across all heterostructure films, and the long-lived process did not exhibit a significantly increased time constant or any discernible pattern (Figure S21). These observations further confirm that the “turnaround” feature and the prolonged excited-state lifetime are closely linked to the differences in the excited-state populations of MoS_2_.

To further explore the distinct time constants associated with the “turnaround” feature in the three heterostructure films, we analyzed the pump fluence-dependent dynamics of the C exciton and PIA-1 in HS-TM. The differential transmittance signals at the “turnaround” for both the C exciton and PIA-1 displayed a linear relationship with varying pump fluence and maintained a constant characteristic time (Figure S22). This finding indicates that the fraction of energy transferred to the MoS_2_ lattice is governed by the heat capacity of each component and the thermal conductance at the interface, rather than by the pump fluence itself. Therefore, we attribute the variations in acoustic phonon recycling across the three heterostructure films to differences in thermal conductance at the interfaces. Compared to HS-TM (Figure 4c), an additional layer of Ti_3_C_2_T_*x*_ in HS-TMT delivers more heat to MoS_2_ while simultaneously offering better insulation, leading to a prolonged characteristic time of ∼100 ps (Figure 4d). HS-TTM, which has a Ti_3_C_2_T_*x*_-Ti_3_C_2_T_*x*_ interface and a Ti_3_C_2_T_*x*_-MoS_2_ interface, exhibits more pronounced differences (Figure 4e). Thermal transport across the Ti_3_C_2_T_*x*_-Ti_3_C_2_T_*x*_ interface is less efficient than that across the Ti_3_C_2_T_*x*_-MoS_2_ interface. This reduced efficiency at the Ti_3_C_2_T_*x*_-Ti_3_C_2_T_*x*_ interface likely contributes to the longer time constant (∼175 ps) observed for HS-TTM (i.e., “tertiary excitation”). Overall, our findings suggest that interfacial engineering through multilayer stacking can effectively modulate the interfacial thermal transport efficiency and affect the time duration of the acoustic phonon recycling process. Such modulation could extend the lifetime of excited states, offering potential benefits for carrier extraction in the related applications.

### Ultrafast Charge/Energy Transfer

**Figure 5:**
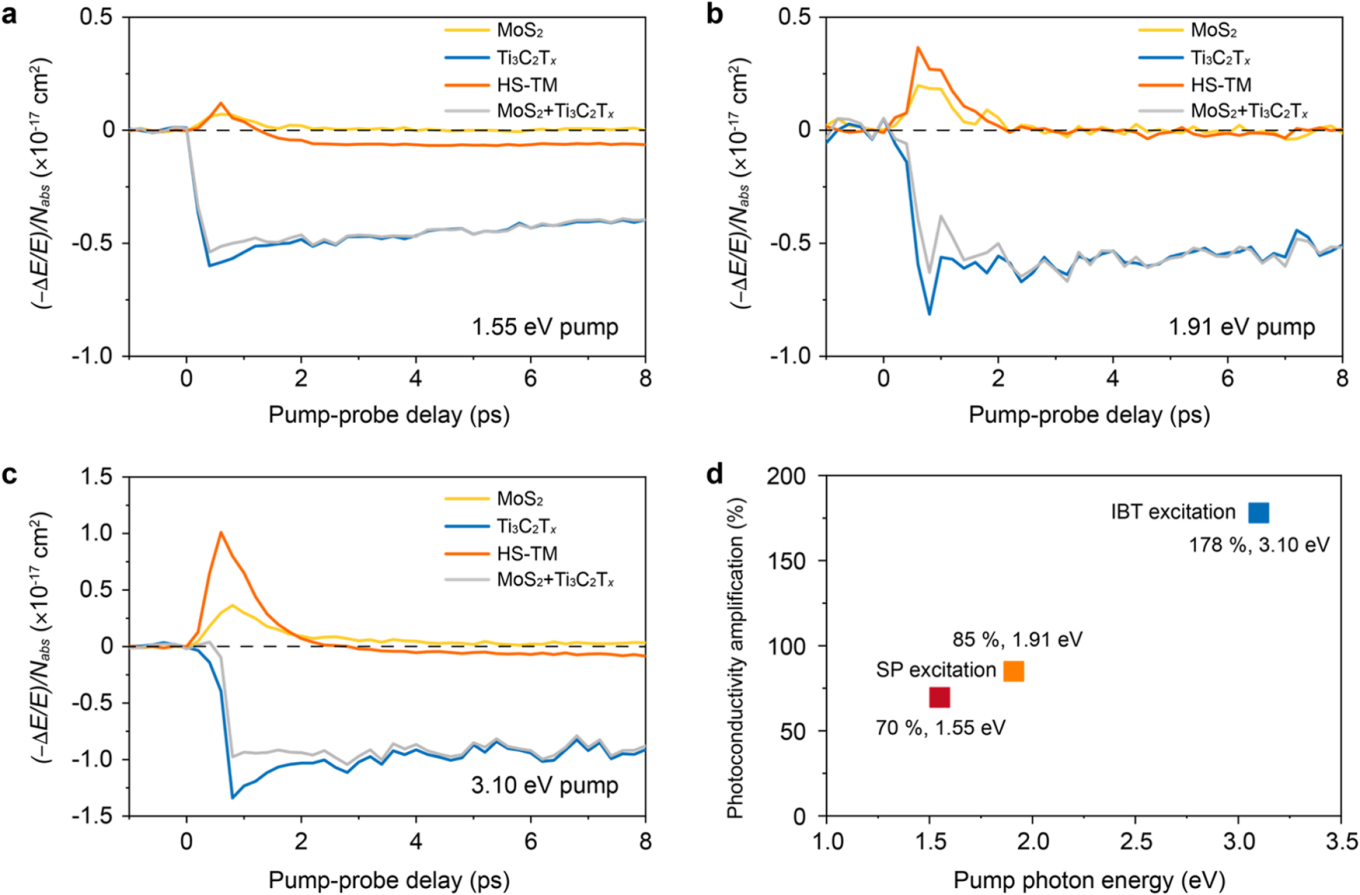
Ultrafast interfacial charge/energy transfer probed by THz spectroscopy. (a–c) THz photoconductivity dynamics normalized by absorbed photon density of the MoS_2_ film, the Ti_3_C_2_T_*x*_ film, HS-TM, and the superimposed responses of the MoS_2_ film and the Ti_3_C_2_T_*x*_ film under (a) 1.55 eV, (b) 1.91 eV, and (c) 3.10 eV excitations. (d) Comparison of photoconductivity enhancement under different excitations.

In the TA spectra of HS-TM, clear intensity variations within the first 1 ps suggest rapid charge/energy transfer at the Ti_3_C_2_T_*x*_-MoS_2_ interface. However, overlapping spectral signals from Ti_3_C_2_T_*x*_ and MoS_2_ complicate the direct extraction of this process. In this context, optical pump-THz probe (OPTP) spectroscopy provides complementary insights by measuring transient conductivity changes via the pump-induced relative change in the transmitted THz electrical field (−Δ*E*/*E* = [*E*_withpump_ – *E*_w/opump_]/*E*_w/opump_).^43,44^Figure 5a compares the photoconductivity dynamics normalized by absorbed photon density ((−Δ*E*/*E*)/*N*_abs_) for the Ti_3_C_2_T_*x*_ film, the MoS_2_ film, and HS-TM under 1.55 eV excitation. The Ti_3_C_2_T_*x*_ film showed a long-lived negative photoconductivity following SP excitation, indicative of heating effects typically observed in metallic systems.^45^ Sub-bandgap excitation at 1.55 eV, below the A exciton resonance of MoS_2_ at 1.88 eV, led to positive photoconductivity in MoS_2_, attributed to defect-assisted free carrier generation.^43^ The resulting photoconductivity decayed rapidly within 2 ps due to the recapture of transient free carriers into in-gap defect states. Intriguingly, HS-TM under 1.55 eV excitation displayed a transient positive photoconductivity that reversed to negative within 2 ps, followed by a long-lived negative photoconductivity plateau persisting for over 10 ps. This dynamic feature deviates from a simple, linearly superimposed response of individual MoS_2_ and Ti_3_C_2_T_*x*_ films, indicating the involvement of interfacial processes in HS-TM. The suppression of negative photoconductivity originating from hot carriers in Ti_3_C_2_T_*x*_, combined with the amplification of positive photoconductivity associated with free carriers in MoS_2_, point to efficient charge/energy transfer from Ti_3_C_2_T_*x*_ to MoS_2_. Furthermore, the instantaneous changes in both TA and THz photoconductivity dynamics of HS-TM, compared to the individual components, suggest that the charge/energy transfer occurs in an ultrafast manner, faster than the instrument response (∼150 fs). Otherwise, HS-TM would exhibit similar TA and THz dynamics with the superimposed responses of individual MoS_2_ and Ti_3_C_2_T_*x*_ films at the initial stage. The ultrafast charge/energy transfer from Ti_3_C_2_T_*x*_ to MoS_2_ enhances the maximum positive photoconductivity of HS-TM, which is dominated by free carriers in MoS_2_, compared to the MoS_2_ film under an equivalent absorbed photon density and identical pump wavelength. To quantify this enhancement resulting from the ultrafast interfacial charge/energy flow from Ti_3_C_2_T_*x*_ to MoS_2_, we defined the photoconductivity amplification ratio, η (%), of HS-TM based on eq 2, where [(−Δ*E*/*E*)/*N*_abs_]_(HS–TM(max))_ and [(−Δ*E*/*E*)/*N*_abs_]_MoS_2_(max)_ represent the maximum photoconductivity per absorbed photon density for HS-TM and the MoS_2_ film, respectively. Based on this definition, the η of HS-TM under 1.55 eV excitation is estimated to be ∼70%.
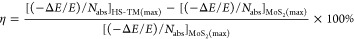
2

To gain further insights into the interfacial charge/energy transfer mechanism, we performed pump-wavelength-dependent OPTP measurement at 1.91 eV (Figure 5b) and 3.10 eV (Figure 5c). In both cases, HS-TM retained the characteristic dynamic features observed under 1.55 eV excitation, namely, suppressed negative photoconductivity compared to Ti_3_C_2_T_*x*_ and enhanced positive photoconductivity relative to MoS_2_. This consistency indicates that the interfacial charge/energy flow is directed from Ti_3_C_2_T_*x*_ to MoS_2_ across all employed pump-photon energies. Notably, the photoconductivity amplification increased with higher excitation photon energy, rising from ∼70% at 1.55 eV to ∼85% at 1.91 eV and reaching ∼180% at 3.10 eV (Figure 5d). This trend suggests that the interfacial charge/energy transfer efficiency increases as the excitation photon energy rises. Such behavior is consistent with the previously reported hot carrier transfer scenario: the excess kinetic energy of hot carriers facilitates interfacial charge separation processes, leading to enhanced transfer efficiency with increasing the pump photon energy.^46,47^ The involvement of hot carriers in ultrafast charge/energy transfer has also been recently observed at the MXene/dye molecule interfaces, including the direct transfer of nonthermalized electrons to molecules and heat transfer by interfacial scattering of nonthermalized electrons.^36^

## Conclusions

In summary, we fabricated a series of heterostructure films with tunable sequences of monolayer Ti_3_C_2_T_*x*_ and MoS_2_ films through an interfacial self-assembly approach. These macro-scale heterostructure films enabled us to directly investigate excited-state dynamics at the interface using pump–probe techniques. Importantly, we discovered a secondary excitation of MoS_2_ in HS-TM under 1.55 eV excitation. This observation is explained by acoustic phonon recycling, where heat generated in Ti_3_C_2_T_*x*_ transfers to MoS_2_ and activates electronic excitation. The trilayer HS-TMT and HS-TTM heterostructure films demonstrated that interfacial thermal transport engineering can extend the excited-state lifetime of MoS_2_, with the HS-TTM configuration exhibiting a time constant exceeding 800 ps. We also observed an ultrafast charge/energy transfer process from Ti_3_C_2_T_*x*_ to MoS_2_ occurring within 150 fs, with the transfer efficiency increasing with higher excitation photon energy. This study advances our comprehension of photogenerated carrier dynamics in the Ti_3_C_2_T_*x*_-MoS_2_ heterostructure and presents promising strategies for controlling excited-state lifetimes in MoS_2_, which is expected to impact the advancement of their photochemical and optoelectronic applications.

## Supporting Information Available

Experimental methods; computational results; optical microscopy, TEM, AFM, STEM, and GIWAXS images; XRD, Raman, and XPS analyses; TDT, and kinetics spectra (PDF)
