# Supplementary material for: Interface-Tailored Secondary Excitation and Ultrafast Charge/Energy Transfer in Ti3C2Tx-MoS2 Heterostructure Films
Source: J Am Chem Soc. 2025 Mar 7;147(11):10012–22. doi: 10.1021/jacs.5c01826 (PMC11926866; doi:10.1021/jacs.5c01826)
Supplement: Supplementary file 1 — ja5c01826_si_001.pdf [file ja5c01826_si_001.pdf]

Supporting information for

Interface-Tailored Secondary Excitation and Ultrafast Charge/Energy Transfer in $Ti_3C_2T_x$-MoS_2 Heterostructure Films

Jiaxu Zhang,[1, ‡] Rafael Muñoz-Mármol,[2, 3, ‡] Shuai Fu,[1, ‡] Xiaodong Li,[1, 4, ‡] Wenhao Zheng,[5] Andrea Villa,[3] Giuseppe M. Paternò,[3, 6] Darius Pohl,[7] Alexander Tahn,[7] Mike Hambsch,[8] Stefan C. B. Mannsfeld,[8] Dongqi Li,[1] Hao Xu,[1] Quanquan Guo,[1] Hai I. Wang,[5, 9, *] Francesco Scotognella,[3, 10, *] Minghao Yu,[1, 4, *] Xinliang Feng[1, 4, *]

[1] Faculty of Chemistry and Food Chemistry & Center for Advancing Electronics Dresden (cfaed), Technische Universität Dresden, 01062 Dresden, Germany

[2] Instituto Universitario de Materiales, University of Alicante, 03690 San Vicente del Raspeig, Spain

[3] Department of Physics, Politecnico di Milano, 20133 Milan, Italy

[4] Max Planck Institute of Microstructure Physics, 06120 Halle (Saale), Germany

[5] Max Planck Institute for Polymer Research, 55128 Mainz, Germany

[6] Center for Nanoscience and Technology, Istituto Italiano di Tecnologia, 20134 Milano, Italy

[7] Dresden Center for Nanoanalysis (DCN), Dresden, Center for Advancing Electronics Dresden (cfaed), TUD Dresden University of Technology, 01062 Dresden, Germany

[8] Center for Advancing Electronics Dresden (cfaed) & Faculty of Electrical and Computer Engineering, TUD Dresden University of Technology, 01062 Dresden, Germany

[9] Nanophotonics, Debye Institute for Nanomaterials Science, Utrecht University, Princetonplein1, 3584 CC Utrecht, The Netherlands

[10] Department of Applied Science and Technology, Politecnico di Torino, Corso Duca degli Abruzzi 24, 10129 Torino, Italy

‡ J. Zhang, R. Muñoz-Mármol, S. Fu, and X. Li contributed equally to this work.

* Corresponding Author: h.wang5@uu.nl; francesco.scotognella@polito.it; minghao.yu@tu-dresden.de; xinliang.feng@tu-dresden.de

EXPERIMENTAL SECTION:

Chemicals. Ti_3AlC_2 (Carbon Ukraine), MoS_2 (particle size ~6 µm, max. 40 µm, Sigma-Aldrich), LiF (≥ 99%, Carl Roth), *n*-butyllithium (1.6 M in hexane, Sigma-Aldrich), ethyl acetate (> 99.5%, TCI), hydrochloric acid solution (≥ 37%, Honeywell Fluka), nitric acid (≥ 65%, Sigma-Aldrich). hexane anhydrous (> 96%, TCI), deionized water (0.055 µS/cm), SiO_2/Si wafer (300 nm SiO_2 coating, thickness = 525 ± 25 µm, MicroChemicals), Si wafer (thickness = 525 ± 25 µm, MicroChemicals), and quartz wafer (thickness = 700 ± 25 µm, MicroChemicals) were bought and directly employed without additional treatment.

Preparation of monolayer $Ti_3C_2T_x$ nanoflakes. 800 mg of LiF was dissolved in 10 mL of 9 M HCl while stirring. Subsequently, 500 mg of Ti_3AlC_2 was slowly added to the solution with stirring at a speed of 350 revolutions per minute (r.p.m.). The mixture was next transferred to an oil bath at 35 °C and stirred at 350 r.p.m. for 24 hours. Afterwards, the solid compound was collected and repeatedly washed with deionized water by centrifugation at 3500 r.p.m. Delamination of the obtained $Ti_3C_2T_x$ was achieved by manual shaking for 15 minutes followed by sonication for 45 minutes. To remove unetched Ti_3AlC_2 and multilayer $Ti_3C_2T_x$, the solution was centrifuged at 5000 r.p.m. for 30 minutes, and this step was repeated three times. Finally, the resulting $Ti_3C_2T_x$ dispersion (~2.7 mg mL^{-1}) was degassed with Ar for 30 minutes and stored in a refrigerator at 4°C.

Preparation of monolayer MoS_2 nanoflakes. 300 mg of bulk MoS_2 was added to 5 mL of 1.6 M *n*-butyllithium in hexane and stirred for 48 h in an Ar-protected glovebox. Afterwards, the solid compound was collected, and the excess *n*-butyllithium was then removed by repeatedly washing with anhydrous hexane three times. The resulting solid compound was subsequently transferred to 45 mL of deionized water, forming a black ink-like dispersion. To accelerate delamination, the solution was manually shaken for 15 minutes, followed by sonication for 15 minutes. The resulting solution was centrifuged at 6000 r.p.m. for 30 minutes to remove un-delaminated and multilayer MoS_2, and this step was repeated three times. Finally, the resulting MoS_2 dispersion (~1.9 mg mL^{-1}) was degassed with Ar for 30 min and stored in a refrigerator at 4 °C.

Self-assembly of macro-scale films. To obtain the $Ti_3C_2T_x$ film, 60 µL of the $Ti_3C_2T_x$ dispersion (~2.7 mg mL^{-1}) was diluted with 40 mL of deionized water. Subsequently, 1 mL of 1 M HNO_3 was added to the diluted dispersion to neutralize the negative charge on the $Ti_3C_2T_x$ surface. Afterwards, 2 mL of ethyl acetate was gradually dropped onto the dispersion surface. After the complete evaporation of ethyl acetate, the $Ti_3C_2T_x$ film

formed on the dispersion surface. The assembly of the MoS_2 film followed a similar procedure, starting with a MoS_2 dispersion (~1.9 mg mL^{-1}). The heterostructure films were prepared by carefully scooping up one film onto another dried film. All the films were annealed in an Ar atmosphere at 300 ℃ for one hour.

Characterizations. The morphology of the films was characterized using an optical microscope (ZEISS), transmission electron microscopy, TEM (JEOL JEM F200C), and AFM (Park NX10 AFM). The cross-section of the heterostructure film was obtained using the FEI Helios 660 SEM/FIB. PXRD patterns were obtained on an X-ray diffractometer (Aeris Benchtop XRD System) using Cu-Kα radiation (λ = 1.5418 Å) at 40 kV and 15 mA. UPS and XPS spectra were collected with Thermo Scientific ESCALAB Xi^+ X-ray Photoelectron Spectrometer (XPS) Microprobe. Ultraviolet-visible-near-infrared (UV−Vis−NIR) absorption spectra were measured with a SHIMADZU UV-3600i Plus UV−Vis−NIR spectrophotometer. Raman and PL spectra were acquired using a WITec alpha 300 R–Raman Imaging Microscope with excitations at 532 nm (*N= 1800 gr/mm*) and 488 nm, respectively. The GIWAXS measurements were performed at beamline SIRIUS at SOLEIL, France. The energy of the beam was 12 keV and it had a size of 70 µm (vertical) × 500 µm (horizontal). The detector was a PILATUS 1M area detector and the sample-to-detector distance was 378.8 mm. The distance and the beam center on the detector were verified using silver behenate. The measurements were performed with a grazing incidence angle of 0.1°and the samples were exposed to the beam for six intervals of 30 seconds each. The data was converted, averaged, and analyzed using WxDiff.

Transient absorption (TA) spectroscopy. Ultrafast TA experiments were performed with a Ti:Sapphire laser (800 nm, 2 kHz, 70 fs duration pulse, 2 mJ). The laser output was split in two beams used to generate the pump and probe pulses. On the one hand, pump pulses were generated with a non-collinear optical parametric amplifier (NOPA) that provides 70-fs duration pulses that can be tuned continuously from 480 nm to 680 nm and 400 nm excitation was generated by frequency doubling the laser fundamental with a BBO plate that provides 90-fs duration pulses.[1] On the other hand, the broadband probe beam was generated by directly focusing the 800-nm-beam into a sapphire plate that provides supercontinuum pulses ranging from 380 to 750 nm. Probe's polarization is adjusted with a $\lambda/2$ waveplate placed before the supercontinuum generation to form a 90° angle with respect to the pump's polarization to refuse scattered light during detection. Then, the pump and probe beams were overlapped on the same spot of the sample (pump diameter was 250 µm), while fluences were adjusted with neutral density filters keeping them low to avoid damaging the sample. The pump-probe delay was set by means of a mechanical delay stage and pump was chopped to 1 kHz. Finally, the transient

transmission spectra were collected with an optical multichannel spectrometer, where the transmittance difference signal (D*T*/*T*) was detected after filtering out scattered light from the pump with a polarizer parallel to the probe.

Optical pump-THz probe (OPTP) spectroscopy. The OPTP setup was powered by a commercial regenerative amplifier, mode-locked Ti: sapphire femtosecond laser, operating at a central wavelength of 800 nm, with a pulse duration of approximately 50 fs and a repetition rate of 1 kHz. During the OPTP measurements, the sample was initially excited with photons of varying wavelengths, generated either through a BiB_3O_3 crystal or a commercial optical parametric amplifier from Light Conversion. Following a specified pump-probe delay, the THz pulse, travelling collinearly with the pump pulse, was transmitted through the sample. The single-cycle THz pulse was produced from the 800 nm pump pulse via optical rectification in a 1 mm-thick ZnTe crystal. The THz electric field in the time domain was then characterized using the electro-optic sampling method.

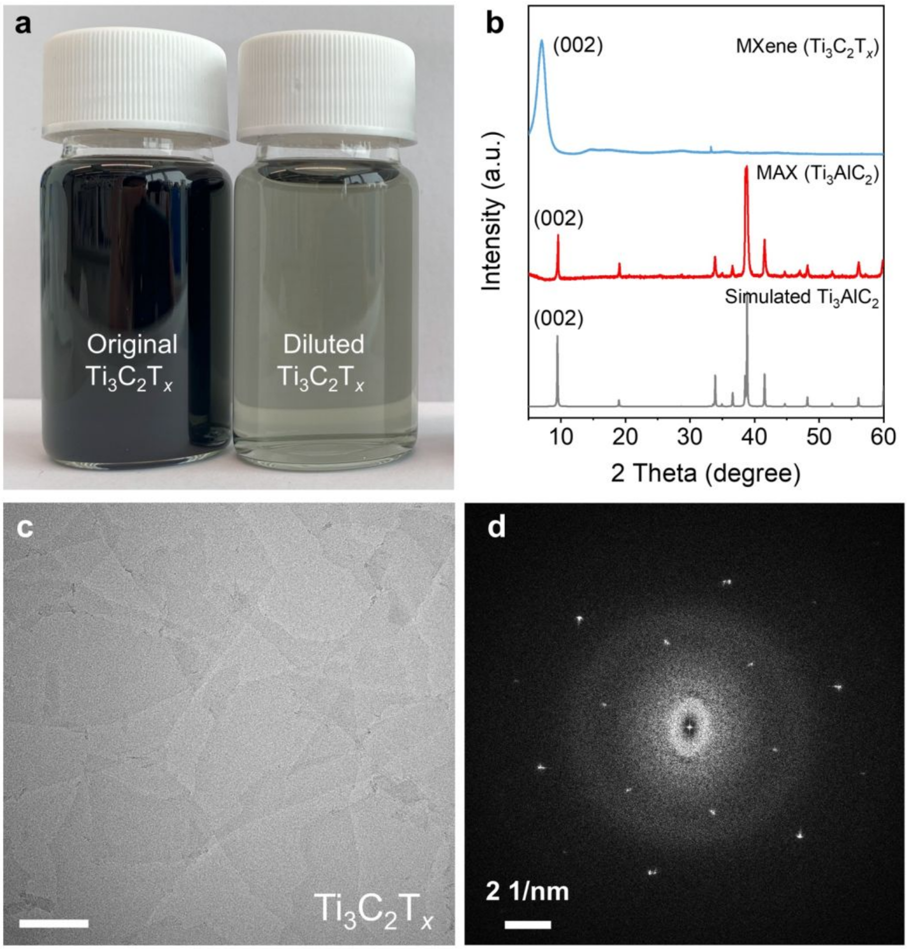

Figure S1. (a) Aqueous dispersions of $Ti_3C_2T_x$. (b) XRD patterns of the Ti_3AlC_2 MAX precursor and the obtained $Ti_3C_2T_x$ MXene. The samples were made by vacuum drying followed by depositing as-prepared aqueous solutions on the SiO_2/Si substrate. (c) TEM image and (d) (calculated, FFT) selected area electron diffraction (SAED) pattern of $Ti_3C_2T_x$ nanoflakes. Scale bar, 100 nm in (c).

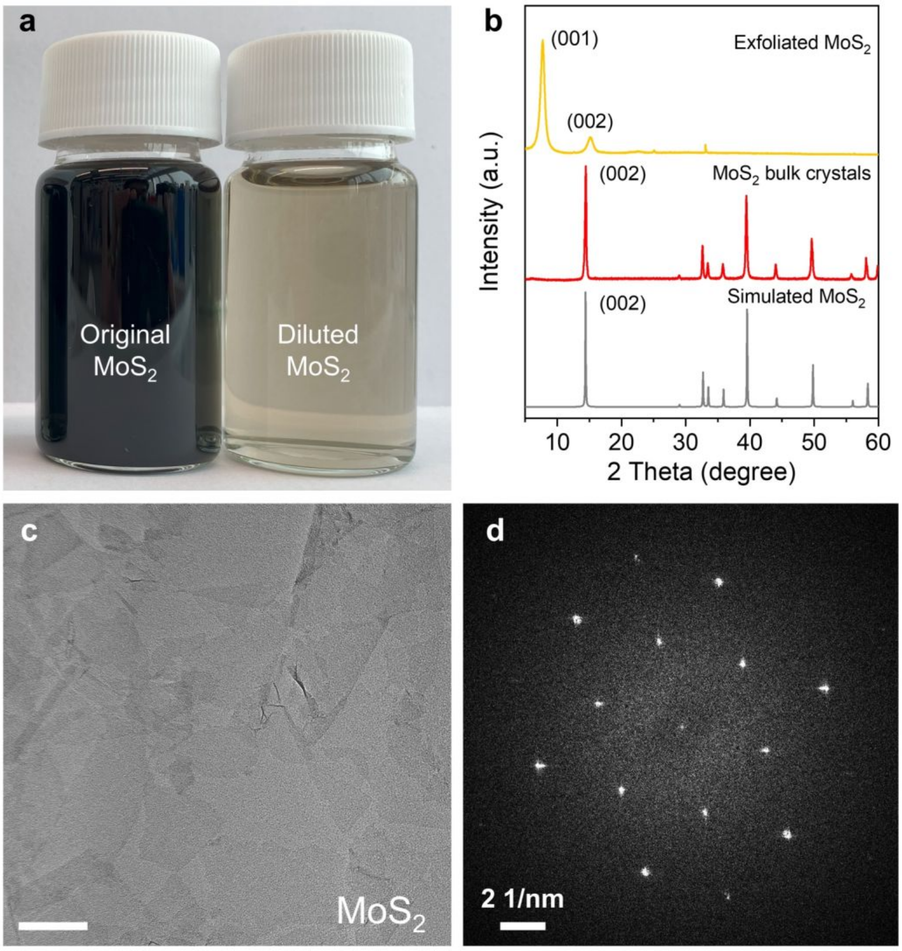

Figure S2. (a) Aqueous dispersions of MoS_2. (b) XRD patterns of the bulk MoS_2 precursor and the exfoliated MoS_2 flakes. The samples were made by vacuum drying followed by depositing as-prepared aqueous solutions on the SiO_2/Si substrate. (c) TEM image and (d) (calculated, FFT) SAED pattern of MoS_2 nanoflakes. Scale bar, 100 nm in (c).

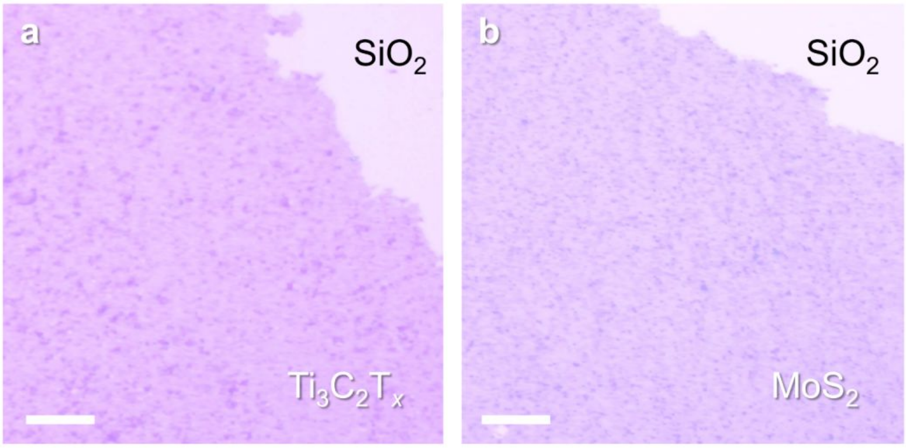

Figure S3. Optical microscopy images of the as-prepared (a) $Ti_3C_2T_x$ film and (b) MoS_2 film. Scale bars, 20 μm.

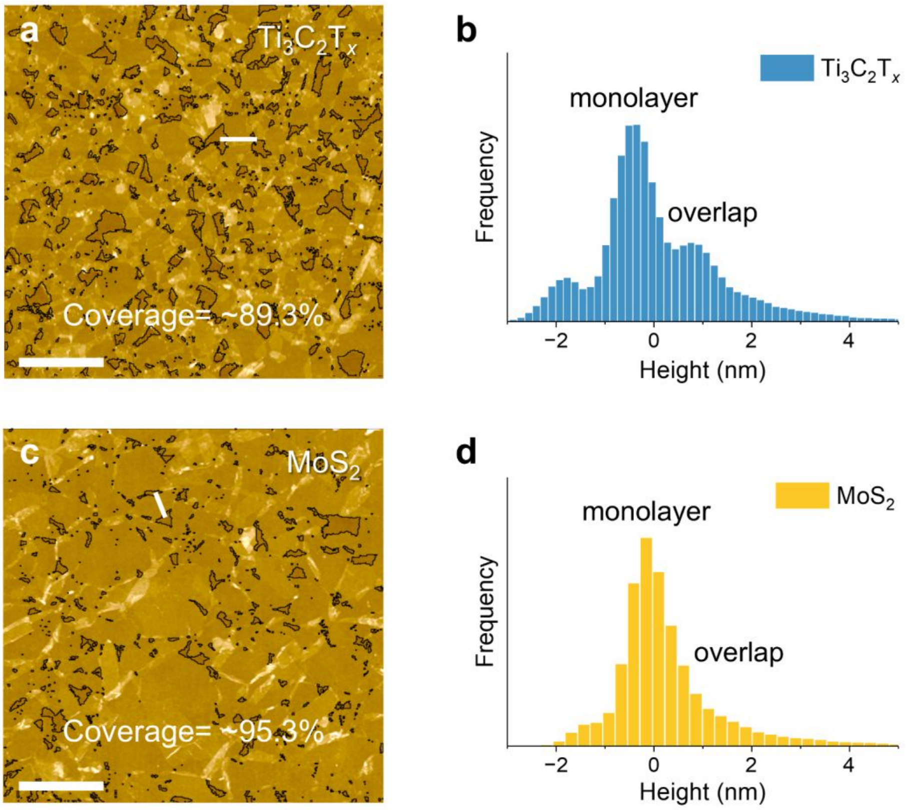

Figure S4. Statistical analysis of coverage and thickness for (a), (b) $Ti_3C_2T_x$ and (c), (d) MoS_2 films, respectively. Scale bars, 1 μm. The thickness distribution in (b) and (d) is derived from the corresponding AFM images in (a) and (c), respectively.

The coverage difference may arise from the intrinsic mechanical properties of MoS_2 and $Ti_3C_2T_x$ nanoflakes, particularly their in-plane stiffness (*C*) and out-of-plane bending rigidity (*D*). MoS_2 nanoflakes exhibit greater inherent flexibility, with lower *C* (123 N/m) and *D* (9.14 eV) values compared to $Ti_3C_2T_x$ nanoflakes (*C*: 214.85 N/m–361.42 N/m, *D*: 28.99 eV–74.29 eV).[2] This flexibility promotes the tendency of MoS_2 nanoflakes to curl, facilitating stacking at boundaries during assembly and leading to higher coverage.

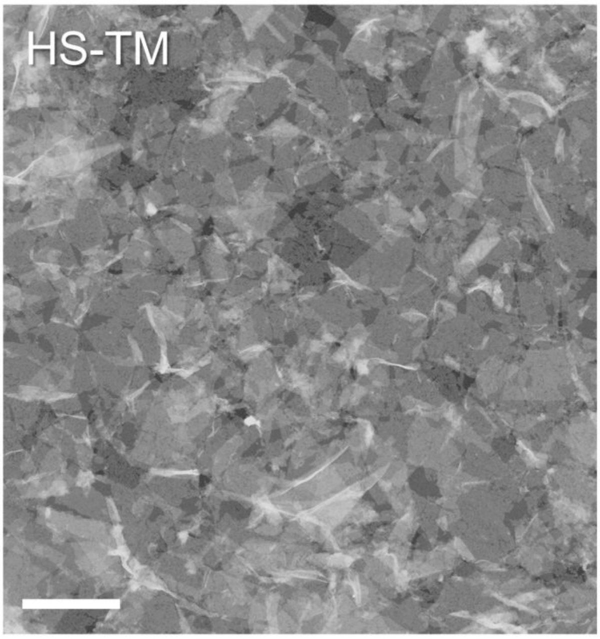

Figure S5. STEM image of HS-TM, scale bar, 500 nm.

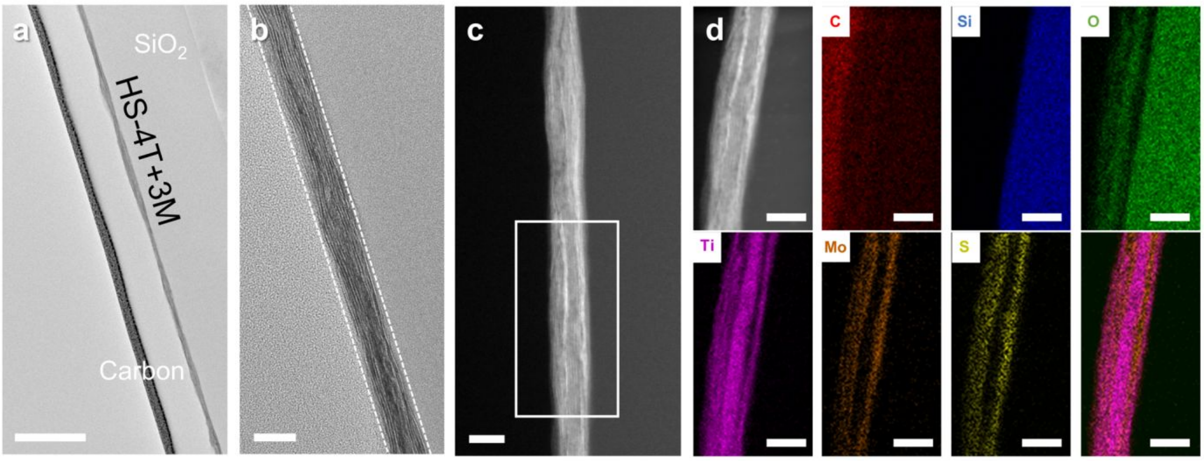

Figure S6. (a), (b) Cross-sectional TEM, (c) STEM and (d) elemental mapping images of a multilayer $Ti_3C_2T_x$-MoS_2 heterostructure film made by alternatively stacking 4 layers of $Ti_3C_2T_x$ film and 3 layers of MoS_2 film. Scale bars, 200 nm in (a), 20 nm in (b), (c) and (d).

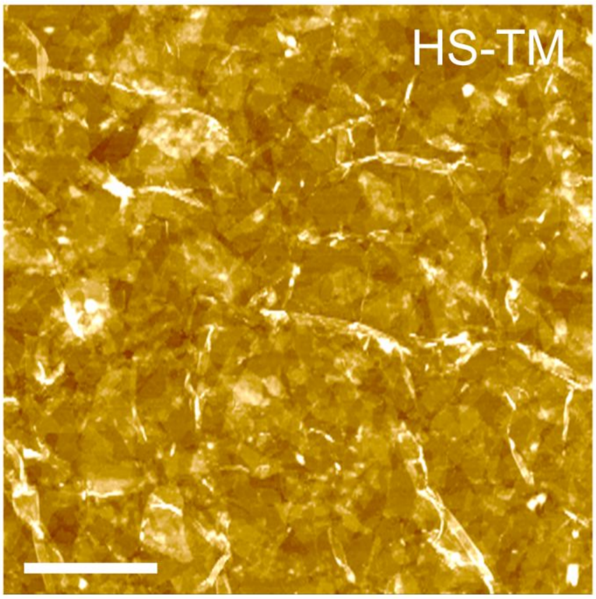

Figure S7. AFM image of HS-TM, scale bar, 1 μm.

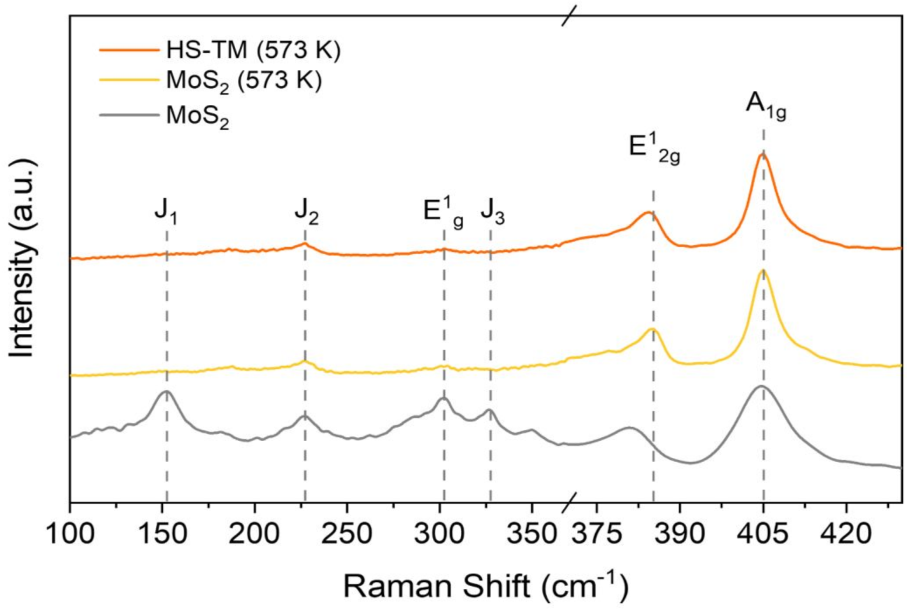

Figure S8. Raman spectra of the as-prepared MoS_2 film, the MoS_2 film after annealing at 300 ℃ and HS-TM. The thermal treatment significantly suppressed the J_1 (~152.3 cm^{-1}), J_2 (~227.0 cm^{-1}) and J_3 (~326.4 cm^{-1}) phonon modes associated with the 1T-MoS_2.[3]

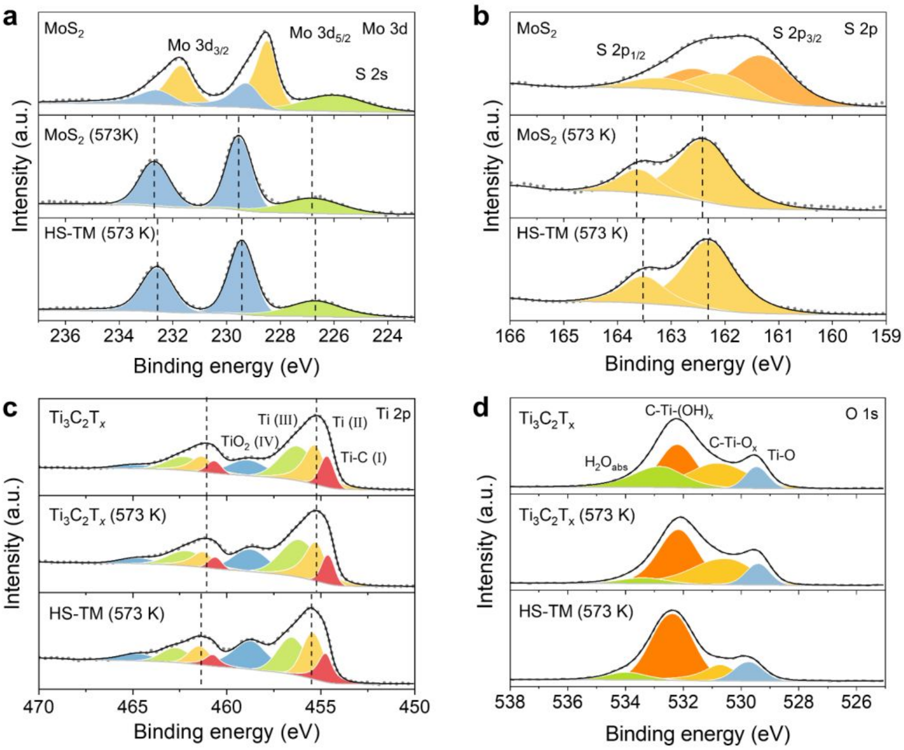

Figure S9. High-resolution (a) Mo 3d and (b) S 2p XPS spectra of the as-prepared MoS_2 film, the MoS_2 film after annealing at 300 ℃ and HS-TM. High resolution (c) Ti 2p and (d) O 1s XPS spectra of the as-prepared $Ti_3C_2T_x$ film, the $Ti_3C_2T_x$ film after annealing at 300 ℃ and HS-TM.

Deconvolution of Mo 3d spectrum from the as-prepared MoS_2 film revealed a distinctive dual-component structure, with higher energy levels corresponding to the 2H phase and lower energy levels corresponding to the 1T phase of MoS_2, respectively.[4] An evident increase in the 2H phase was observed after annealing, leading to a discernible shift towards higher energy levels by ~1 eV, while no significant oxidation was observed, confirming its good stability. Meanwhile, the Ti 2p and O 1s XPS spectra indicate that the $Ti_3C_2T_x$ film demonstrates good thermal stability with only slight oxidation.

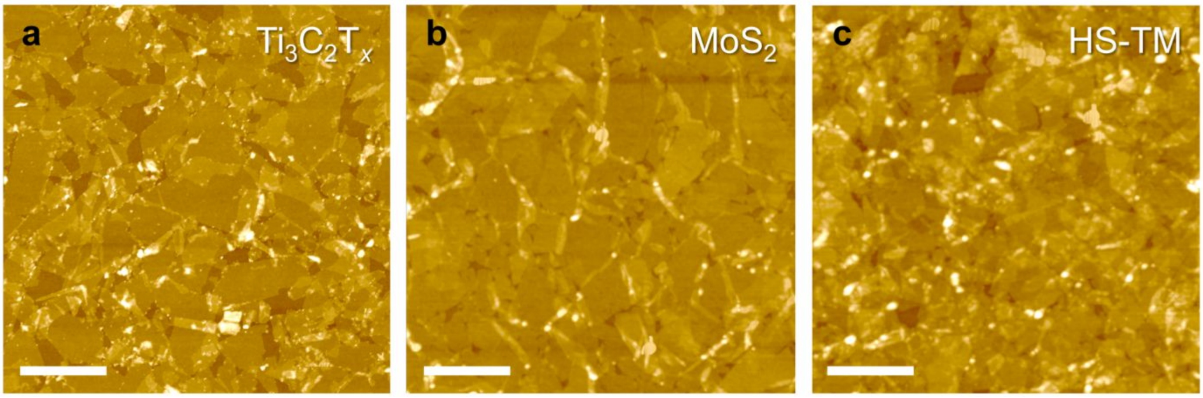

Figure S10. AFM images of (a) $Ti_3C_2T_x$ film (b) MoS_2 film and (c) HS-TM after thermal treatment. Scale bars, 1 μm.

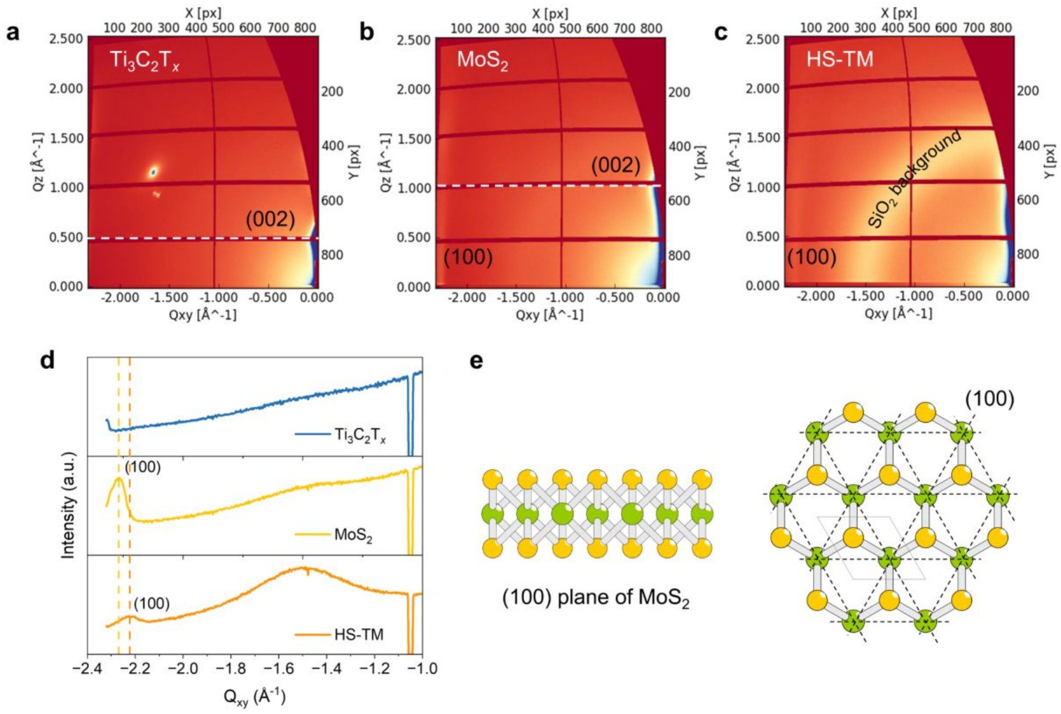

Figure S11. 2D GIWAXS images of (a) the $Ti_3C_2T_x$ film, (b) the MoS_2 film, and (c) HS-TM. (d) In-plane line-cut profiles derived from the GIWAXS patterns. (e) Schematic of the (100) plane of MoS_2.

All distinctive scattering spots observed along the q_z axis are ascribed to lattice planes aligned parallel to the substrate surface. Specifically, the spot observed for the $Ti_3C_2T_x$ film at $q \sim 0.5$ Å^{-1} corresponds to the (002) diffraction.[5] For the MoS_2 film, the spot at $q \sim 1$ Å^{-1} corresponds to the (002) diffraction of the 2H phase.[6] Those interlayer diffractions are attributed to the overlap of flakes and the possible presence of few-layer nanoflakes. Regarding the in-plane scattering signals: we observe a shift of the (100) diffraction signal from $q_{xy} \sim 2.27$ Å^{-1} for MoS_2 to $q_{xy} \sim 2.22$ Å^{-1} for HS-TM. This suggests an in-plane expansion of the lattice upon forming the heterostructure possibly caused by lattice matching between the two constituent crystal structures and indicates interfacial interactions between the two materials in the heterostructure.

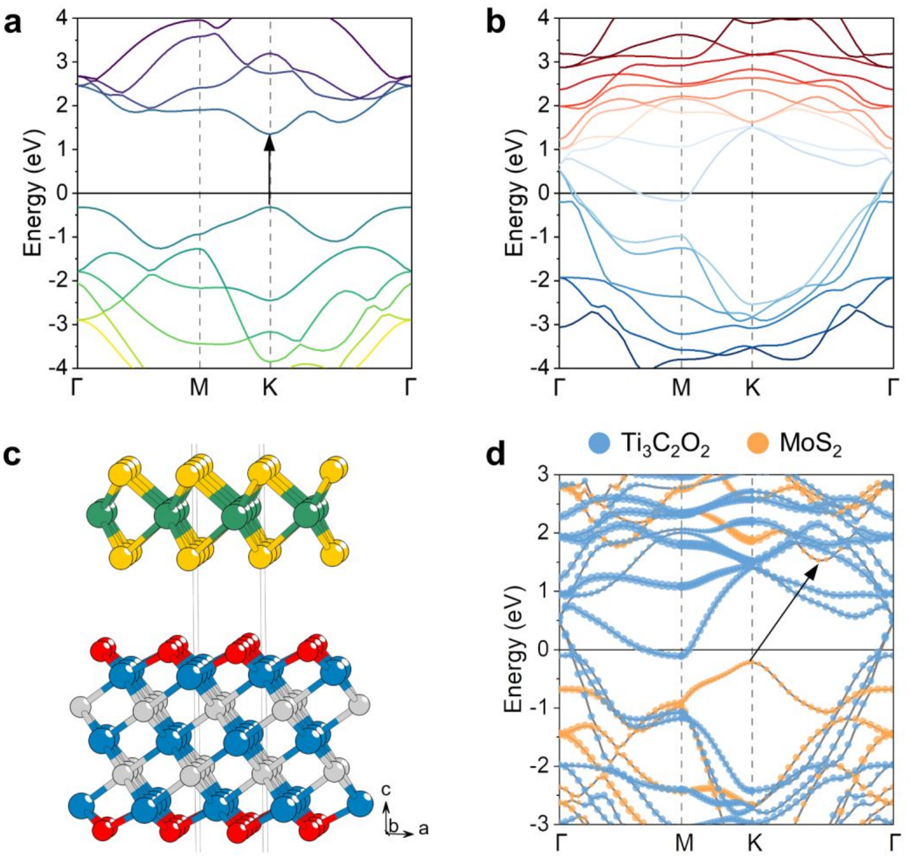

Figure S12. Band structures of (a) MoS_2 and (b) $Ti_3C_2O_2$. (c) The atomic structure of the MoS_2-$Ti_3C_2O_2$ heterostructure, which was used as the model for calculations. (d) Projected band structure of the MoS_2-$Ti_3C_2O_2$ heterostructure.

Given the complex terminations of $Ti_3C_2T_x$, we chose $Ti_3C_2O_2$ as a reference to make the calculations feasible. This choice was based on previous reports indicating that the surface of $Ti_3C_2T_x$ is primarily occupied by O terminations.[7] We calculated the independent band structures of $Ti_3C_2O_2$ and MoS_2 for subsequent comparison. The results showed that $Ti_3C_2O_2$ exhibits metallic characteristics, whereas MoS_2 is characterized by a direct bandgap at the K point of the Brillouin zone.[8,9] In the MoS_2-$Ti_3C_2O_2$ heterostructure, MoS_2 transitions from a direct band gap at the K point to an indirect band gap.

The first-principles calculations were carried out with the Vienna ab initio simulation package (VASP 5.4.4).[10, 11] The interaction between ions and valence electrons is described using projector augmented wave (PAW) potentials, and the exchange-correlation between electrons is treated through using the generalized gradient approximation (GGA) in the Perdew-Burke-Ernzerhof (PBE) form.[12] DFT-D3 method was employed to calculate the van der Waals (vdW) interaction.[13] The parameters of dipole correction were applied for the calculation of slab models. Electronic energies were computed with the tolerance of 1×10^{-5} eV and total force

of 0.01 eV/Å. A kinetic cutoff energy of 450 eV was adopted. The crystal lattice parameters of MoS_2 bulk are as follows: $a = b = 3.18938$ Å, $c = 14.00497$ Å ($\alpha = \beta = 90^{\circ}$, $\gamma = 120^{\circ}$); while the corresponding parameters for $Ti_3C_2O_2$ are $a = b = 3.03951$ Å, $c = 19.45795$ Å ($\alpha = \beta = 90^{\circ}$, $\gamma = 120^{\circ}$). For MoS_2- $Ti_3C_2O_2$ heterostructures, MoS_2 and $Ti_3C_2O_2$ monolayers along (001) direction are combined to simulate the heterostructure. The initial structure is obtained after computing their single point energy of heterostructure with MoS_2 slabs shifting along z direction. To achieve the accurate simulations, a Gamma centered k-point grid of 7 × 7 × 2 and 3 × 3 × 1 was used for bulk and slab models.

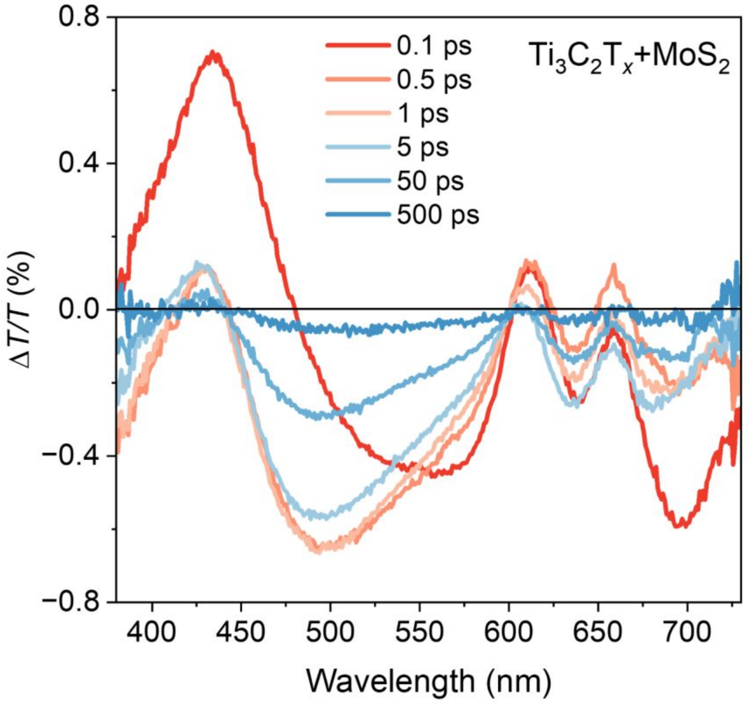

Figure S13. Superposed TDT spectra of the $Ti_3C_2T_x$ film and the MoS_2 film in a weight ratio of 1:1.

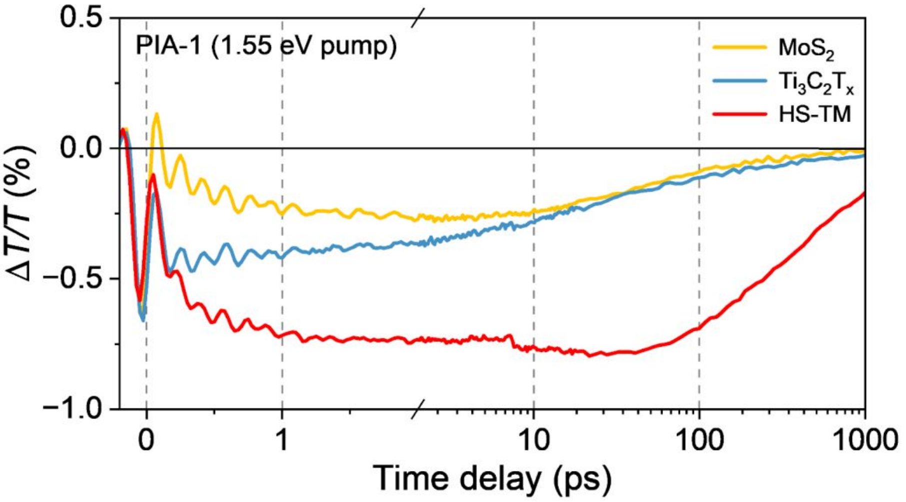

Figure S14. TA kinetics of PIA-1 for the MoS_2 film, the $Ti_3C_2T_x$ film, and HS-TM under 1.55 eV excitation.

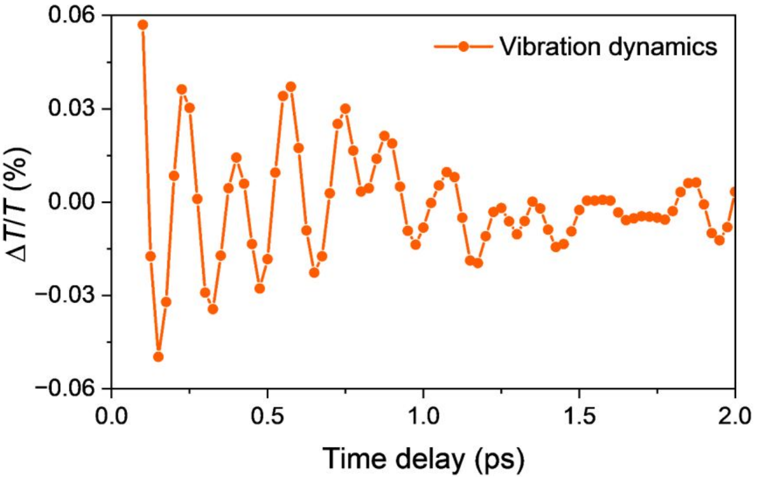

Figure S15. The vibration dynamics extracted from the kinetic curve of PIA-1 for HS-TM under 1.55 eV excitation.

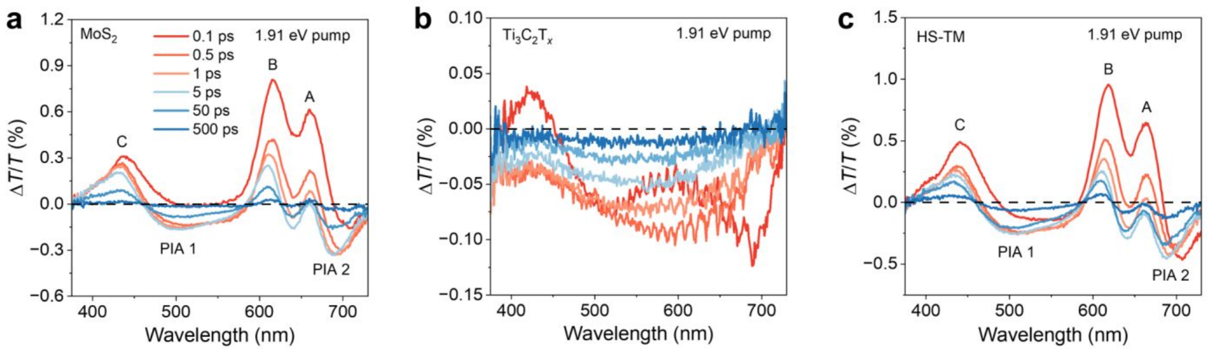

Figure S16. TDT spectra of (a) the MoS_2 film, (b) the $Ti_3C_2T_x$ film, and (c) HS-TM at 0.1 ~ 500 ps under 1.91 eV excitation.

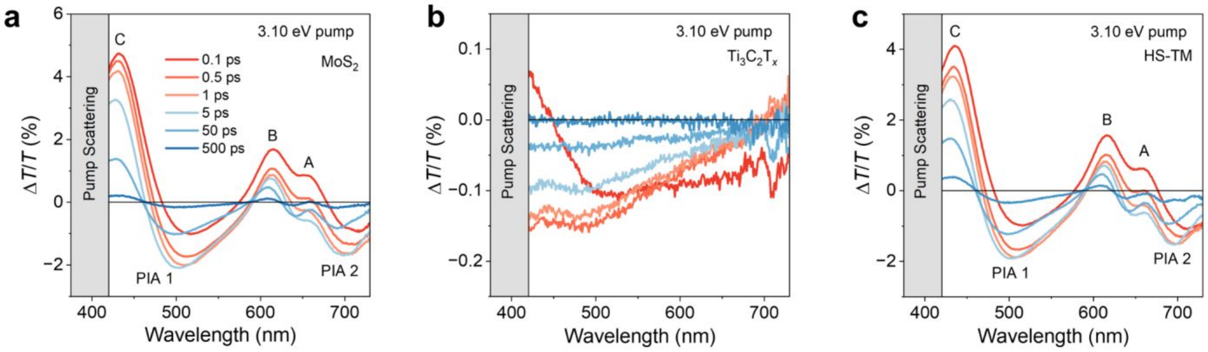

Figure S17. TDT spectra of (a) the MoS_2 film, (b) the $Ti_3C_2T_x$ film, and (c) HS-TM at 0.1 ~ 500 ps under 3.10 eV excitation.

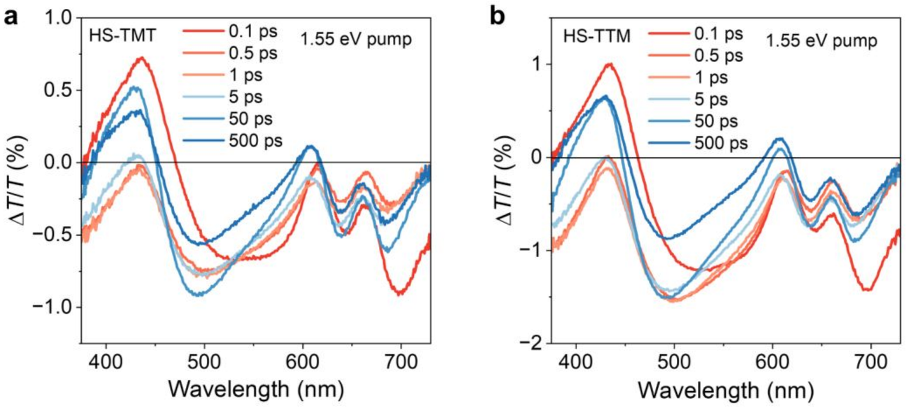

Figure S18. TDT spectra of (a) HS-TMT and (b) HS-TTM at 0.1 ~ 500 ps under 1.55 eV excitation.

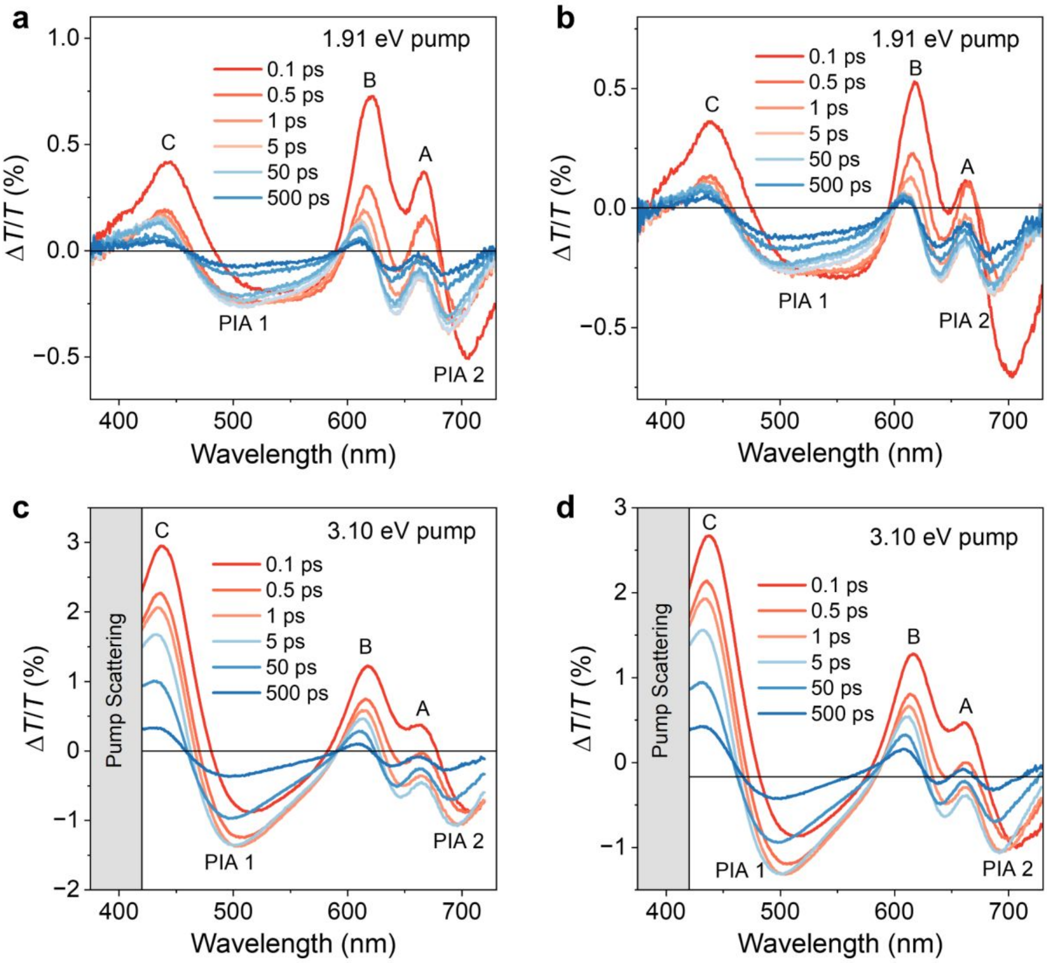

Figure S19. TDT spectra of (a) HS-TMT and (b) HS-TTM at 0.1 ~ 500 ps under 1.91 eV excitation. TDT spectra of (c) HS-TMT and (d) HS-TTM at 0.1 ~ 500 ps under 3.10 eV excitation.

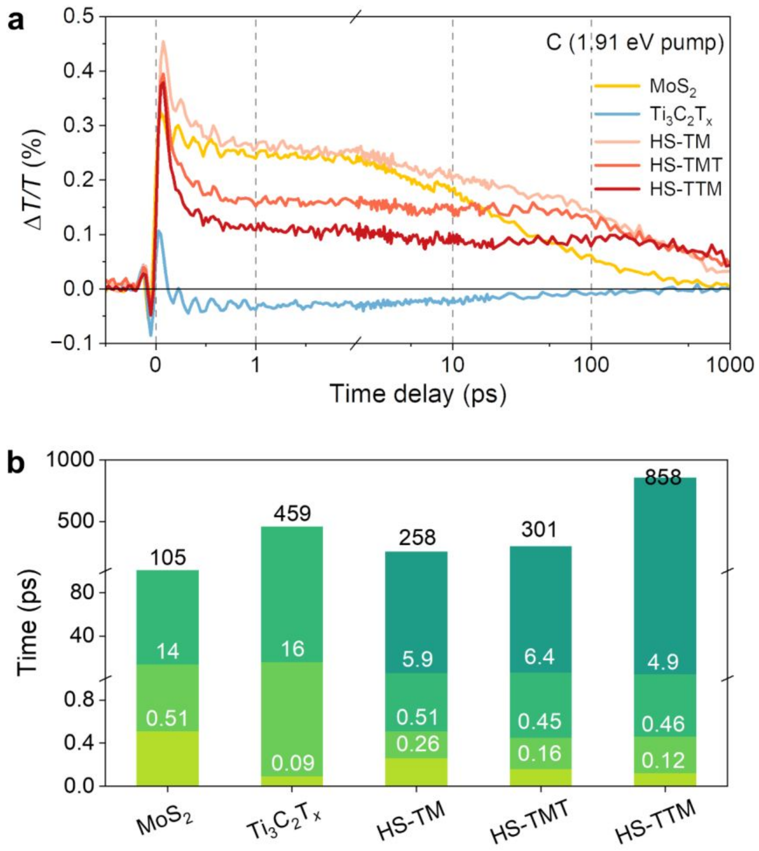

Figure S20. (a) TA kinetics and (b) the corresponding exponentially fitted time constants of the MoS_2 film, the $Ti_3C_2T_x$ film, HS-TM, HS-TMT, and HS-TTM probed at 436 nm (C exciton bleaching) under excitation at 1.91 eV.

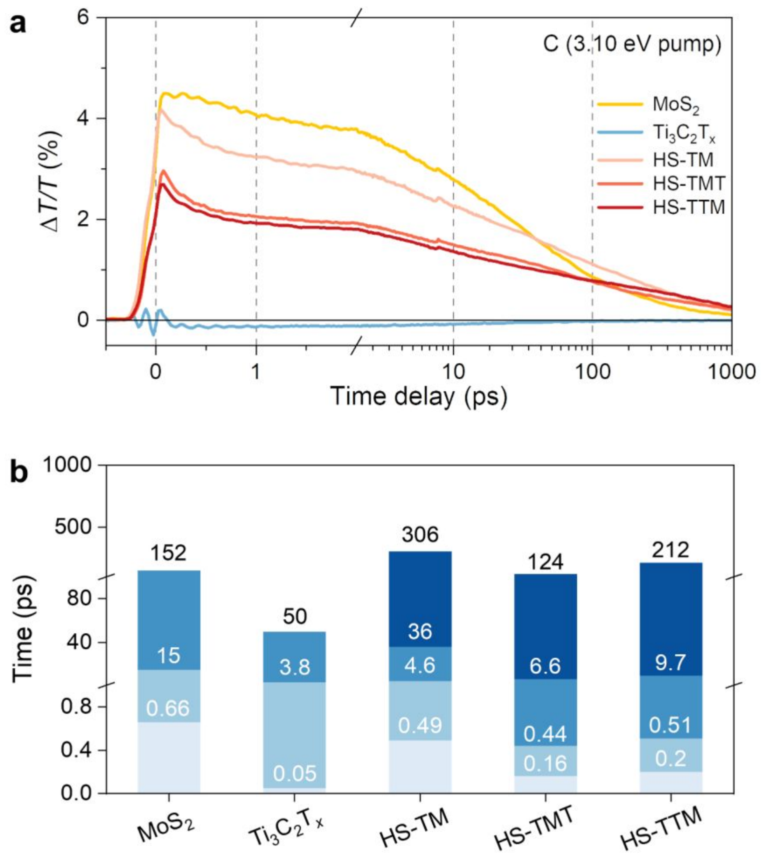

Figure S21. (a) TA kinetics and (b) the corresponding exponentially fitted time constants of the MoS_2 film, the $Ti_3C_2T_x$ film, HS-TM, HS-TMT, and HS-TTM probed at 436 nm (C exciton bleaching) under excitation at 3.10 eV.

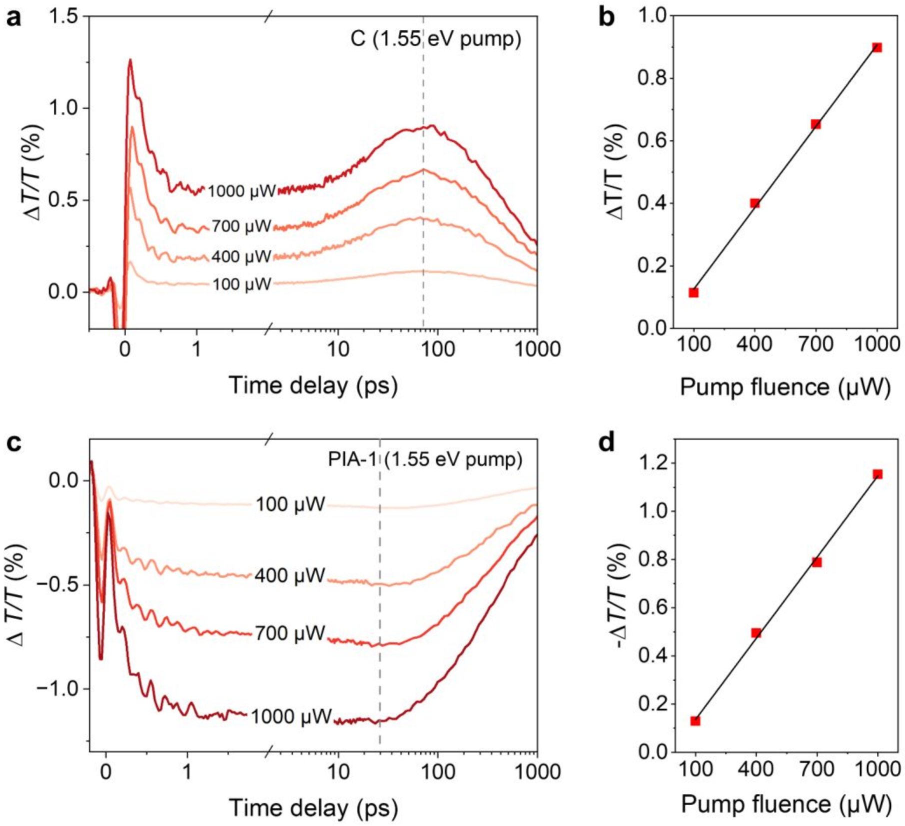

Figure S22. Pump fluence-dependent dynamics of HS-TM, probed at (a) C exciton bleaching and (c) PIA-1 under 1.55 eV excitation, with the intensities of (b) C exciton bleaching and (d) PIA-1 measured at the 'turnaround' as a function of pump fluence.

References

1. Manzoni, C.; Cerullo, G. Design criteria for ultrafast optical parametric amplifiers. *J. Opt.* **2016**, *18*, 103501.

2. Hu, T.; Yang, J.; Li, W. et al. Quantifying the rigidity of 2D carbides (MXenes). *Phys. Chem. Chem. Phys.* **2020**, *22*, 2115–2121.

3. Nayak, A. P. et al. Pressure-dependent optical and vibrational properties of monolayer molybdenum disulfide. *Nano Lett.* **2015**, *15*, 346–353.

4. Eda, G. et al. Photoluminescence from chemically exfoliated MoS_2. *Nano Lett.* **2011**, *11*, 5111–5116.

5. Qin, L. et al. A flexible semitransparent photovoltaic supercapacitor based on water-processed MXene electrodes. *J. Mater. Chem. A* **2020**, *8*, 5467–5475.

6. Sojková, M. et al. Tuning the orientation of few-layer MoS_2 films using one-zone sulfurization. *RSC Adv.* **2019**, *9*, 29645–29651.

7. Hope, M. A. et al. NMR reveals the surface functionalisation of Ti_3C_2 MXene. *Phys. Chem. Chem. Phys.* **2016**, *18*, 5099–5102.

8. Splendiani, A. et al. Emerging photoluminescence in monolayer MoS_2. *Nano Lett.* **2010**, *10*, 1271–1275.

9. Ma, S. et al. Exploring the catalytic activity of MXenes $M_{n+1}C_nO_2$ for hydrogen evolution. *J. Mater. Sci.* **2019**, *54*, 11378–11389.

10. Kresse, G.; Furthmüller, J. Efficiency of ab-initio total energy calculations for metals and semiconductors using a plane-wave basis set. *Comp. Mater. Sci.* **1996**, *6*, 15–50.

11. Surendranath, Y.; Kanan, M. W.; Nocera, D. G. Mechanistic studies of the oxygen evolution reaction by a cobalt-phosphate catalyst at neutral pH. *J. Am. Chem. Soc.* **2010**, *132*, 16501–16509.

12. Perdew, J. P.; Burke, K.; Ernzerhof, M. Generalized gradient approximation made simple. *Phys. Rev. Lett.* **1996**, *77*, 3865–3868.

13. Grimme, S.; Antony, J.; Ehrlich, S.; Krieg, H. A consistent and accurate abinitio parametrization of density functional dispersion correction (DFT-D) for the 94 elements H-Pu. *J. Chem. Phys.* **2010**, *132*, 154104.
